# Salidroside-loaded stem cell-derived artificial nanovesicles in hydrogel microneedles alleviate inflammation and enhance diabetic wound regeneration

**DOI:** 10.1016/j.mtbio.2026.103323

**Published:** 2026-06-05

**Authors:** Junhao Xia, Fengya Wang, Yang Song, Yu Xu, Mengru Zhu, Wenkun Sun, Yaqi Zhang, Sichun Wang, Qingwen Zhang, Keman He, Xin Guan, Hanhan Zhang, Xiulin Wang, Lukuan Liu, Jing Liu

**Affiliations:** Stem Cell Clinical Research Center, The First Affiliated Hospital of Dalian Medical University, Dalian, 116011, China

**Keywords:** Diabetic wound healing, Hydrogel microneedles, Stem cell-derived artificial nanovesicles, Salidroside

## Abstract

Diabetic wounds often arise from a dysregulated wound microenvironment characterized by persistent inflammation, impaired angiogenesis, and aberrant macrophage polarization, leading to delayed healing and severe disability. In this study, we fabricated a stem cell-derived artificial nanovesicles-salidroside@gelatin methacryloyl/polyvinyl alcohol composite microneedle system (CNV-Sa@GelMA/PVA-MN) composed of a GelMA/PVA hydrogel matrix integrated with salidroside loaded stem cell-derived artificial nanovesicles to achieve efficient therapy for diabetic wound. The system penetrated the skin barrier for precise delivery, with stem cell-derived artificial nanovesicles enabling sustained salidroside release and collectively enhancing its anti-inflammatory and pro-angiogenic activities in vitro. Mechanistically, network pharmacology, molecular dynamics simulations, and proteomics revealed the system suppressed the NF-κB pathway, promoted macrophage M1 to M2 polarization, and alleviated inflammatory responses. In vivo experiments confirmed it significantly accelerated wound closure and inhibited inflammatory factor release. These findings highlighted a promising strategy for diabetic wound therapy through inflammatory microenvironment regulation and coordinated tissue repair.

## Graphical abstract

**Figure:**
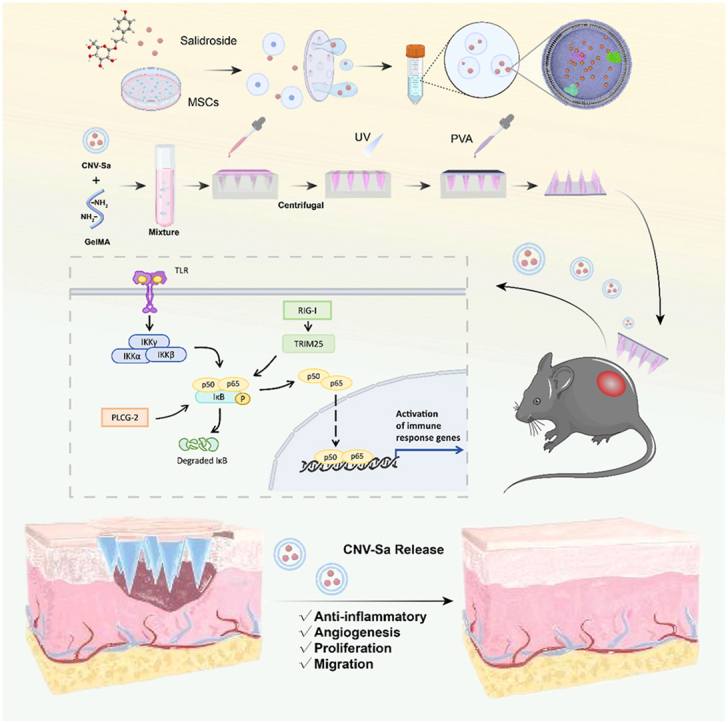

## 1 Introduction

Diabetic patients were prone to developing chronic, non-healing wounds or ulcers, which remained one of the most challenging problems in clinical practice [1]. These wounds were typically characterized by significantly delayed healing, persistent inflammation, and impaired angiogenesis, all of which severely affected patients’ quality of life and imposed a substantial global healthcare burden [2]. In a hyperglycemic microenvironment, sustained metabolic dysregulation induced cellular senescence and immune imbalance, particularly disrupting the normal polarization of macrophages [3,4]. During normal wound healing, dynamic phenotypic switching of macrophages was a critical process that drove the transition of wounds from the inflammatory phase to the proliferative and remodeling phases [5,6]. However, in diabetic wounds, this polarization process was markedly impaired, leading to persistent accumulation of pro-inflammatory M1 macrophages, prolonged expression of inflammatory mediators, and sustained chronic inflammation, which collectively hindered effective wound repair [7,8]. Therefore, developing multifunctional therapeutic strategies capable of systemically modulating aberrant macrophage polarization to alleviate delayed healing, persistent inflammation, and impaired angiogenesis represented a highly promising direction for diabetic wound treatment.

Mesenchymal stem cell (MSC) derived vesicles could replicate the therapeutic effect of MSCs on diabetic wounds and avoiding the side effects associated with stem cell transplantation [9]. But the large-scale production of vesicle-based therapies remained highly challenging. The average daily yield of vesicles from 10^6^ cells was only 1-4 μg, which failed to meet the dosage requirements for clinical trials (100-500 μg per patient) [10]. Recent studies showed that nanovesicles obtained from parental cells via the extrusion method possessed distinct advantages over vesicles, including a 250-fold higher production yield and a twofold increase in the content of RNAs and proteins, as well as remarkable antioxidant capacity and potent mitochondrial function restoration ability [11,12]. Notably, our research group found that stem cell-derived artificial nanovesicles (CNVs) prepared by extrusion were enriched in mitochondrial active proteins and exhibited favorable potential in radiation-induced skin injury [13] and diabetic wound [14]. However, the persistent and dysregulated inflammatory microenvironment characteristic of diabetic wounds posed substantial challenges to achieving optimal therapeutic efficacy. Therefore, enhancing the anti-inflammatory functionality of CNVs while preserving their intrinsic biological properties emerged as a critical strategy for improving their therapeutic performance. To this end, researchers attempted to load active ingredients into stem cell-derived artificial nanovesicles to optimize their performance. Such functional nano-delivery systems could compensate for inherent defects [15], significantly enhanced anti-inflammatory and pro-angiogenic capabilities, and provided a novel solution for the precise repair of diabetic wounds.

Salidroside (Sa) was a bioactive compound derived from *Rhodiola rosea*, a plant that grows at high altitudes [16]. Studies demonstrated that Sa possessed various pharmacological activities, including antioxidant [17], anti-inflammatory [18], anti-stress [19], etc. Structurally, Sa was a phenolic compound composed of a tyrosol molecule conjugated with a glucose molecule [20]. From a physicochemical perspective, Sa exhibited high aqueous solubility but limited membrane permeability, which might lead to rapid diffusion and clearance from the wound surface following topical administration [21]. However, diabetic wounds were typically deep, making it difficult for Sa to penetrate the lower tissue layers, which limited its therapeutic efficacy. To prolong its retention time and enhance tissue penetration, an appropriate delivery strategy was needed. In this research, CNVs served as an ideal carrier for Sa encapsulation, providing sustained and targeted drug release while utilizing the intrinsic pro-regenerative and anti-inflammatory properties of CNVs to synergistically promote wound healing.

During topical wound delivery, the skin barrier system significantly hindered the penetration of active substances, thereby reducing therapeutic efficacy [22]. Microneedle patches, as multifunctional, portable, and minimally invasive delivery systems, could effectively bypass the stratum corneum and enable targeted transport of drugs, nanoparticles, and other therapeutic agents into deeper tissues or even the systemic circulation [[23], [24], [25]]. They not only overcame the limitations of insufficient penetration associated with conventional topical delivery but also minimized pain and tissue damage [26]. In this study, microneedles were fabricated via a mold-casting strategy, which is universal, facile, and compatible with diverse hydrogel systems and functional payloads. GelMA/PVA-MN offered excellent mechanical strength, biocompatibility, and tunable degradation properties. Their microscale architecture enabled efficient tissue adaptation and spatiotemporally drug release [27]. In addition, the PVA maintained appropriate flexibility, making GelMA/PVA-MN an ideal carrier for delivering therapeutic nanocomposites into complex wound environments [28]. This facile and scalable fabrication approach endows the current microneedle system with favorable potential for clinical translation.

In this study, a microneedle array based on a composite hydrogel of GelMA and PVA was constructed, and CNVs loaded with Sa were embedded therein to successfully prepare a novel CNV-Sa@GelMA/PVA-MN therapeutic system. This system exhibited multi-level coordinated therapeutic properties. On one hand, CNVs served as ideal delivery carriers for Sa, enabling the sustained release of Sa, avoiding its rapid loss on the wound surface, and significantly improving the retention time and bioavailability of Sa locally at the wound. On the other hand, relying on its inherent anti-inflammatory and pro-angiogenic activities, Sa could significantly enhance the inherent pro-regenerative function of CNVs, and facilitated the resolution of chronic inflammation in diabetic wounds. Meanwhile, the GelMA/PVA composite matrix endowed the microneedles with excellent mechanical strength and controllable degradation performance, allowing them to efficiently penetrate the skin stratum corneum barrier and accurately deliver the loaded therapeutic components to the deep tissues of diabetic wounds. This engineered microneedle system achieved the integrated coordination of efficient delivery and multi-dimensional pro-healing effects, providing a novel system for the treatment of chronic diabetic wounds.

## 2 Materials and methods

### 2.1 Cell culture

The human umbilical cord mesenchymal stem cells (hUCMSCs) used in this study comply with the principles of the Declaration of Helsinki, with the informed consent of the pregnant women and family members and were approved by the Ethics Committee of the First Hospital of Dalian Medical University (registration number: PJ-KS-KY-2023-97). The AMMS^Ⓡ^MSC Kit 2.0 (Tongli Haiyuan, Beijing,China) provided nutritional support for the cells. Human skin fibroblasts (HSF), human keratinocytes (HaCaT), and human umbilical vein endothelial cells (HUVEC) were purchased from Zhong Qiao Xin Zhou (Shanghai, China). HaCaT and HSF were cultured in DMEM (high glucose) (Zhong Qiao Xin Zhou) supplemented with 10% FBS (Gibco) and 1% penicillin-streptomycin (OriCell^Ⓡ^), while HUVEC were cultured in Endothelial Cell Medium (ScienCell). All cells were cultured under standard sterile conditions at 37 °C with 5% CO_2_, and subcultured when the cell confluence reached 80-90%.

### 2.2 Preparation of CNVs

hUCMSCs were collected, washed once with phosphate buffered saline (PBS) pre-cooled to 4 °C, resuspended for cell counting, and adjusted to a final concentration of 1 × 10^6^ cells/mL. The cell suspension was sequentially extruded through 10 μm, 5 μm, and 1 μm polycarbonate membranes (Whatman) at a constant speed using a liposome extruder, with 6 reciprocating extrusions per membrane. The resulting mixture was centrifuged at 2000×g for 15 min at 4 °C, and the supernatant was collected and filtered through a 0.22 μm filter. The filtrate was further centrifuged at 20,000×g for 30 min at 4 °C, and the precipitate was resuspended in PBS and stored at −80 °C to obtain blank CNVs.

### 2.3 Preparation of salidroside-loaded nanovesicles (CNV-Sa)

hUCMSCs at confluence were digested, centrifuged at 1000 rpm for 5 min, and washed once with pre-cooled 4 °C 1×PBS. Cells were resuspended and counted to adjust the concentration to 1 × 10^6^ cells/mL. Salidroside was dissolved in pre-cooled 1×PBS, then mixed with cell suspension to obtain a solution at a concentration of 10 mg/mL. The mixture was incubated on ice to facilitate the interaction between salidroside and cell membranes. Under ice-bath conditions, the mixture was extruded using a liposome extruder following the same membrane pore sizes and reciprocation parameters as described in Section 2.2. All subsequent centrifugation, filtration, ultracentrifugation, pellet rinsing, resuspension and storage procedures were also performed in accordance with Section 2.2.

### 2.4 Determination of drug loading performance

CNV-Sa were lysed with 1% Triton X-100, and salidroside was quantified by ultraviolet-visible spectrophotometry (UV-Vis) at a detection wavelength of 274 nm. The standard curve was established with a linear range of 30-250 μg/mL, showing an excellent linear relationship with a correlation coefficient (*R*^2^) of 0.999 (Fig. S1). All subsequent samples were quantified based on this standard curve.

Loading capacity and drug loading content were calculated using the following Equations:

$$Loading\;Capacity\;\left(\%\right)=\frac{W_{loading}}{W_{CNV-Sa}+W_{loading}}\times 100\%$$

$$Drug\;Loading\;Content\;\left(\mu g/\mu g\right)=\frac{W_{loading}}{W_{CNV-Sa}}$$

Where $W_{loading}$ is the weight of loaded Sa, $W_{CNV-Sa}$ is the total weight of CNV-Sa.

### 2.5 Cell culture under high-glucose conditions

Cells were routinely cultured in DMEM (high glucose). To examine cell-related biological functions under high-glucose conditions, an additional glucose solution was added to the culture medium, resulting in a final glucose concentration of 35 mM [29].

### 2.6 Establishment of the inflammation model

We established an inflammation model using human monocytic leukemia cells (THP-1), which were cultured in specialized medium without the need for trypsin digestion during passaging. The model was established in two steps: first, THP-1 cells were treated with 100 ng/mL phorbol 12-myristate 13-acetate (PMA) for 24 h to differentiate them into M0 macrophages; then, after removing PMA and washing with PBS, the cells were further cultured for 24 h in medium containing 1 μg/mL lipopolysaccharide (LPS) to induce inflammation. During treatment, CNV-Sa and LPS were added simultaneously. After culture, the medium was collected for ELISA analysis (temporarily stored at −20 °C), and the cells were harvested for qRT-PCR experiments (Table S1).

### 2.7 Preparation of GelMA and GelMA/PVA-MN

GelMA was synthesized by dissolving 10 g A-type porcine skin gelatin in 100 mL PBS at 55 °C, followed by dropwise addition of 8 mL methacrylic anhydride and vigorous stirring at 60 °C for 3 h. The reaction was quenched with fourfold volume of PBS (40 °C) and stirred for 30 min. The solution was dialyzed against distilled water for 7 days using a 12-14 kDa MWCO membrane and lyophilized to obtain porous GelMA, stored at −80 °C. GelMA solutions (10-30%) containing 0.5% 2-Hydroxy-4′-(2-hydroxyethoxy)-2-methyl-propiophe (I2959) photoinitiator and 20% PVA solution were prepared. Conical microneedle molds were fabricated via 3D printing, and PDMS negative molds were obtained after vacuum degassing and curing. GelMA solution was filled into the PDMS molds, centrifuged to ensure complete needle filling, followed by addition of PVA as the base and UV curing (365 nm, 3 min). Microneedles were dried at 33 °C overnight and demolded, yielding GelMA/PVA-MN with needle tips containing 10%, 20%, or 30% GelMA.

### 2.8 Construction of full-thickness skin defect model in diabetic mice

To establish the model, first induce Type I diabetes (Ethics Approval Number: AEE24266). Select 6-8-week-old, 18-20 g male C57BL/6J mice, acclimate for 1 week with normal feeding, then fast for 12-14 h (with water). Weigh mice and intraperitoneally inject a freshly prepared 1% (w/v) streptozotocin (STZ) solution at 120 mg/kg. Monitor blood glucose via tail vein sampling on days 1-3 and day 7, identifying mice with polydipsia, polyphagia, polyuria, weight loss, and blood glucose>16.7 mmol/L on day 7 as diabetic. Then, anesthetize diabetic mice with 10 mL/kg of 1% pentobarbital sodium, remove dorsal hair, and create an 8 mm diameter full-thickness skin defect using a biopsy punch. The mice were divided into the following groups: 1) Control group. 2) Diabetic wound model group (DW). 3) Blank GelMA/PVA-MN group (MNs). 4) CNV@GelMA/PVA-MN group (MN-CNVs). 5) Sa@GelMA/PVA-MN group (MN-Sa). 6) CNV-Sa@GelMA/PVA-MN group (MN-CNV-Sa). 7) Free CNV-Sa group. Wound photographs were taken on days 0, 3, 7, 10 and 14. H&E staining was conducted on tissues from day 7 and day 14, and Masson staining was conducted on day 14 tissues. On day 14, wound tissues were collected for immunofluorescence staining of IL-6, IL-1β and immunohistochemical staining of CD31. Major organs including heart, liver, spleen, lung and kidney were harvested for H&E staining.

### 2.9 Data analysis

All experiments were repeated at least three times, and the data are presented as mean ± standard deviation (SD). Differences between two independent samples were analyzed using the independent samples *t*-test, while comparisons among multiple groups were conducted using one-way analysis of variance (ANOVA) followed by Tukey's post hoc test. Statistical significance was evaluated using GraphPad Prism software (v10.1.2), with differences considered statistically significant when *p* < 0.05, ∗*p* < 0.05, ∗∗*p* < 0.01, ∗∗∗*p* < 0.001, ∗∗∗∗*p* < 0.0001.

## 3 Results and discussion

### 3.1 Fabrication and characterization of CNV-Sa

**Fig. 1:**
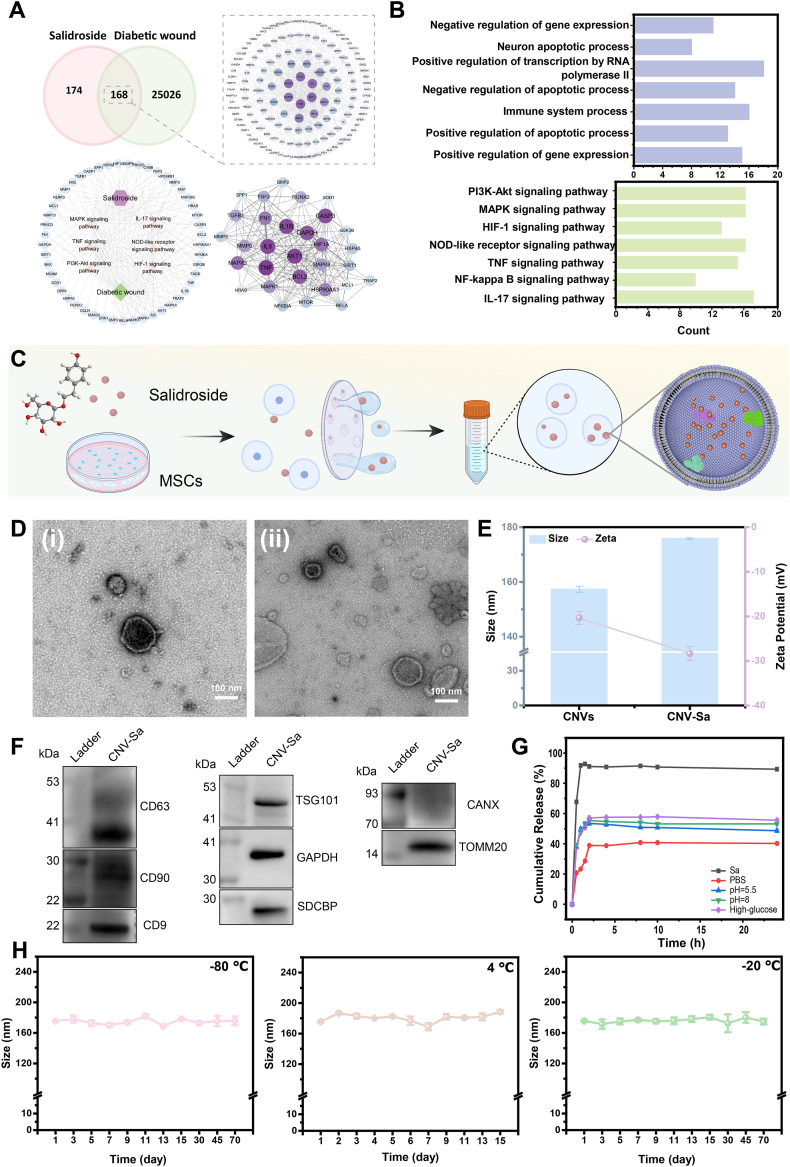
(A) Predicted drug-target-disease network of salidroside and diabetic wound. (B) GO biological process enrichment and KEGG pathway analysis of key signaling pathways regulated by predicted key genes. (C) Scheme for the preparation of CNV-Sa. Created with BioRender.com. (D) Morphology of CNVs (i) and CNV-Sa (ii). Scale bars is 100 nm. (E) Particle size and zeta potential of CNVs and CNV-Sa. (F) Western blot analysis of extracellular vesicle marker proteins (CD63, CD9, CD90, TSG101, GAPDH, SDCBP) and other proteins (CANX, TOMM20) in CNV-Sa. (G) In vitro cumulative release profiles of free Sa and CNV-Sa under different microenvironmental conditions. (H) Particle size stability of CNV-Sa stored at −80 °C, 4 °C and −20 °C over time.

Salidroside possessed potent anti-inflammatory, pro-angiogenic and tissue-repairing activities [30], and exerted promising therapeutic effects on diabetic wounds. Network pharmacology analysis yielded 168 core overlapping targets via the intersection of diabetic wound-associated disease targets and potential molecular targets of Sa (Fig. 1A). Subsequent Gene Ontology (GO) functional enrichment and Kyoto Encyclopedia of Genes and Genomes (KEGG) pathway enrichment analyses were performed using the DAVID database (Fig. 1B), which demonstrated that these core targets were significantly enriched in key signaling pathways including PI3K-Akt, MAPK, HIF-1, TNF and NF-κB all of which are directly implicated in core wound-healing biological processes such as cell survival, inflammatory modulation and angiogenesis.

On this basis, hUCMSCs served as parental cells. Both CNVs and Sa-incorporated CNV-Sa were fabricated by continuous mechanical extrusion based on membrane self-assembly to strengthen anti-inflammatory and pro-repair effects in diabetic wound repair (Fig. 1C, Fig. S2). The drug loading content and loading capacity of CNV-Sa were determined to be 0.938 ± 0.067 μg/μg and 48.35 ± 1.83%, respectively. Based on our previous work on CNVs yield [14], we further quantified the production efficiency of CNV-Sa. As shown in Figure S3, 2.30 ± 0.12 × 10^11^ CNV-Sa particles with a total protein content of 205.36 ± 4.13 μg were harvested from 1.5 × 10^7^ hUCMSCs, verifying the high yield and good reproducibility of the CNV-Sa preparation protocol. Transmission electron microscopy showed that after loading Sa, the morphology of CNV-Sa remained stable and had the same bilayer membrane "cup-shaped" structure (Fig. 1D). Particle size analysis showed a slight increase in the size of CNV-Sa compared to non-loaded CNVs. Its zeta potential decreased with a concomitant rise in absolute value, leading to enhanced vesicle stability. (Fig. 1E). Western blot analysis further detected the protein markers of CNV-Sa. The positive expression of vesicle-associated proteins (CD63, CD9, TSG101 and SDCBP) indicated that CNV-Sa possessed vesicle-related properties. The detection of CD90 reflected the parental MSC origin of these nanovesicles [31]. TOMM20 (mitochondria-associated protein) and CANX (endoplasmic reticulum-associated protein) were also detected in CNV-Sa. The presence of these intracellular compartment proteins supports the general cell-derived characteristic of mechanically extruded nanovesicles (Fig. 1F).

Subsequently, we investigated the in vitro release kinetics of free Sa and CNV-Sa. Free Sa exhibited an obvious burst release behavior under physiological pH 7.4 (PBS). In contrast, CNV-Sa remarkably alleviated the initial burst release and presented a more moderate release pattern, confirming the effective retention of bioactive drugs by CNVs nanovesicles. In the acidic microenvironment at pH 5.5 that mimics the pathological condition of diabetic wounds [32], the release rate of CNV-Sa was significantly accelerated. Similarly, alkaline condition (pH 8) and high-glucose environment could also regulate its drug release behavior, showing distinct microenvironment-dependent release characteristics (Fig. 1G). Storage stability analysis showed minimal changes in particle size under different storage conditions (−80 °C, 4 °C, and −20 °C), indicating good stability of CNV-Sa (Fig. 1H). In addition to temperature stability, we further assessed the structural integrity of CNV-Sa in simulated physiological environments and during lyophilization. When incubated in 10% FBS at 37 °C for 7 days, CNV-Sa exhibited only minor fluctuations in particle size (Fig. S4A). Moreover, comparison of particle size before and after lyophilization showed that rehydrated CNV-Sa maintained the size distribution (Fig. S4B). Degradation assays demonstrated that CNV-Sa group exhibited significantly prolonged stability with a markedly reduced degradation rate (Fig. S4C). These findings demonstrate that CNV-Sa remains stable not only in serum-containing environments but also during freeze-drying and reconstitution, supporting its suitability for long-term storage and formulation development.

### 3.2 Functional evaluation of CNV-Sa in regulating cellular activity

**Fig. 2:**
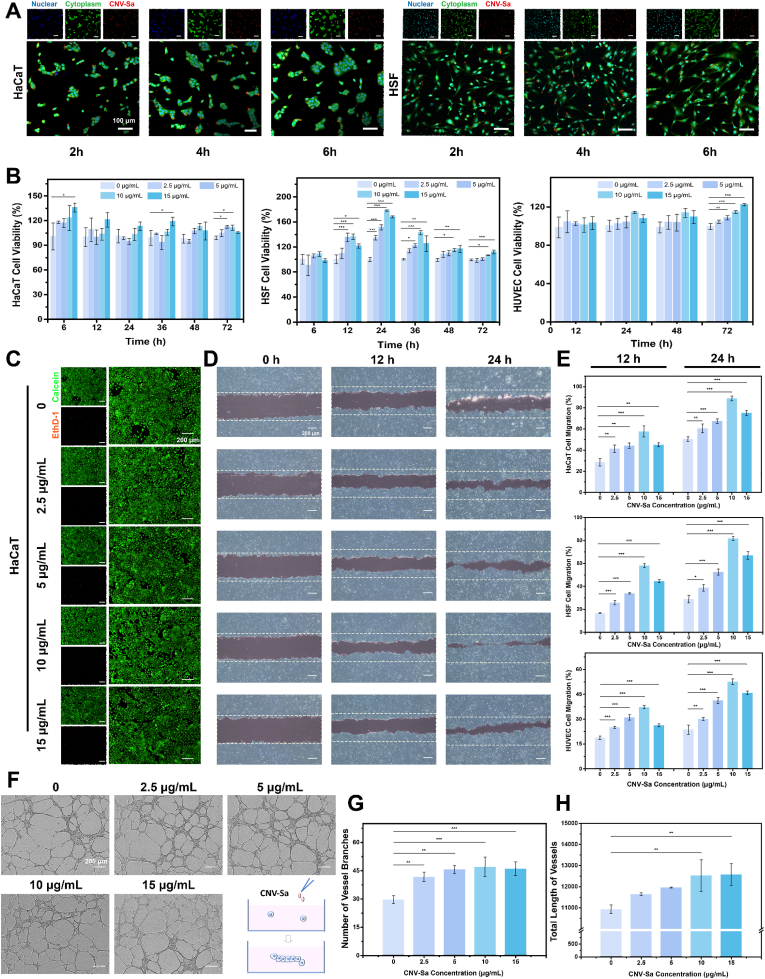
(A) Time-dependent uptake of CNV-Sa by HaCaT and HSF. Scale bar is 100 μm. (B) Quantitative evaluation of the effects of CNV-Sa on the viability of HaCaT, HSF, and HUVEC cells. (C) Live/dead staining of HaCaT after CNV-Sa treatment. Scale bar is 200 μm. (D) Migration images of HaCaT treated with different concentrations of CNV-Sa. Scale bar is 200 μm. (E) Quantification of cell migration in HaCaT, HSF, and HUVEC at different CNV-Sa concentrations. (F) Vascularization of HUVEC treated with CNV-Sa. Scale bar is 200 μm. (G) Quantification of vessel branch numbers. (H) Quantification of total tube length of vessels. All n = 3, ∗*p* < 0.05, ∗∗*p* < 0.01, ∗∗∗*p* < 0.001, ∗∗∗∗*p* < 0.0001.

To elucidate the functional role of CNV-Sa in wound repair, we systematically evaluated its effects on cellular uptake, cell migration, angiogenesis, and viability. After co-incubation of PKH26-labeled CNV-Sa for 2 h, red fluorescence was detected in the cytoplasm of HaCaT and HSF (Fig. 2A). Furthermore, intracellular fluorescence increased significantly over time, demonstrating efficient, time-dependent internalization of CNV-Sa. To further dissect the underlying mechanism, we performed inhibitor-based endocytic pathway assays. CCK-8 assays first confirmed that 5-(N-ethyl-N-isopropyl)-amiloride (EIPA), methyl-β-cyclodextrin (MβCD), and chlorpromazine (CPZ) at working concentrations had no significant cytotoxicity in HaCaT or HSF cells (Fig. S5A and B), ruling out viability-related confounding effects. Under both normal and high-glucose conditions, flow cytometry showed that only the clathrin-mediated endocytosis inhibitor CPZ significantly reduced CNV-Sa internalization in both cell types (Fig. S5C–F), while EIPA and MβCD had minimal effects. These results indicate that CNV-Sa is primarily internalized via clathrin-dependent endocytosis, a mechanism largely preserved under pathological high-glucose conditions. We next evaluated the cytocompatibility of CNV-Sa via CCK-8 assay. As shown in Fig. 2B, the cell viability of HaCaT, HSF, and HUVEC cells remained above 90% at all tested concentrations (2.5-15 μg/mL) of CNV-Sa during 6-72 h of co-culture, demonstrating excellent cytocompatibility. Live/dead fluorescence staining further confirmed these findings (Fig. 2C), showing that CNV-Sa at all tested concentrations (2.5, 5, 10, and 15 μg/mL) did not induce significant cell death in HaCaT, HSF, or HUVEC over 48 h (Fig. S6). We further evaluated the effects of CNV-Sa on the migratory behavior of different cell types. The results showed that cell migration capacity progressively increased with higher concentrations of CNV-Sa (Fig. 2D, Fig. S7). Statistical analysis revealed that in 24 h, HaCaT, HSF, and HUVEC achieved maximum migration rates of 88.86 ± 2.30%, 81.69 ± 1.80% and 52.54 ± 1.75% respectively at 10 μg/mL (Fig. 2E). These results indicated that CNV-Sa could promote cell migration and survival at various concentrations and had potential as a therapeutic system for wound healing and tissue repair.

Diabetic wounds were commonly characterized by insufficient blood supply and impaired angiogenesis, which were major factors contributing to delayed wound healing and the formation of chronic ulcers, and remained key challenges in current treatments. In this study, endothelial cell tube formation assays were used to evaluate the effects of different concentrations of CNV-Sa on the ability of endothelial cells to form tubular structures (Fig. 2F). The results showed that CNV-Sa significantly promoted endothelial tube formation in a concentration-dependent manner. At concentrations of 2.5-15 μg/mL, both the number of branches and the total length of tubular structures were markedly increased, whereas at higher concentrations (Fig. 2G and H). Taken together, these results demonstrate that CNV-Sa effectively enhanced cellular activity, migration, and regenerative functions, providing strong support for its development as a promising therapeutic system for chronic diabetic wound repair.

### 3.3 Therapeutic efficacy of CNV-Sa in high-glucose microenvironment

**Fig. 3:**
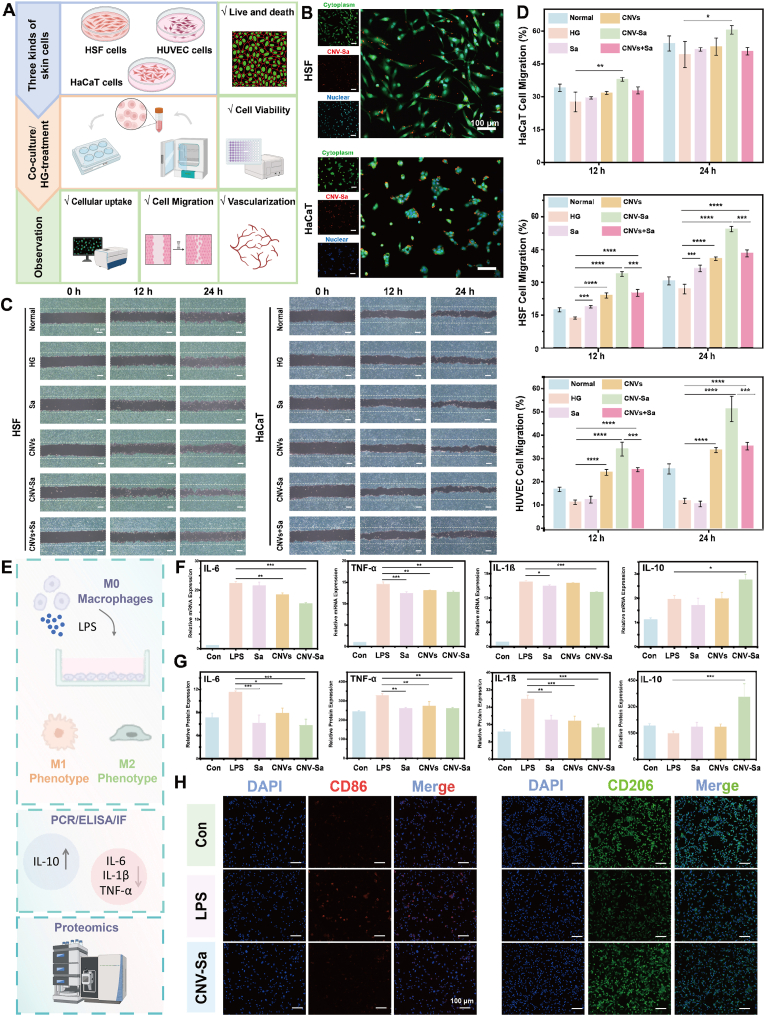
(A) Schematic diagram co-culture of cells with CNV-based therapies under HG condition and evaluation of cellular behaviors. Created with BioRender.com. (B) The in vitro uptake of CNV-Sa by HaCaT and HSF within 2 h under high glucose. Scale bar is 100 μm. (C) Migration capacity of HSF and HaCaT incubated with CNV-based therapies under high glucose conditions. Scale bar is 200 μm. (D) Quantitative analysis of HaCaT, HSF, and HUVEC cell migration under high glucose at 12 h and 24 h after incubated with CNV-based therapies. (E) Scheme of the LPS-induced macrophage inflammatory model and CNV formulations. Created with BioRender.com. (F) qRT-PCR analysis showing the mRNA expression of IL-6, TNF-α, IL-1β and IL-10 in each group. (G) ELISA measurement of IL-6, TNF-α, IL-1β and IL-10 protein levels after different treatments. (H) Immunofluorescence staining of macrophage phenotype markers CD86 (M1 marker, red) and CD206 (M2 marker, green) in each group. Nuclei were counterstained with DAPI (blue). Scale bar is 100 μm. All n = 3, ∗*p* < 0.05, ∗∗*p* < 0.01, ∗∗∗*p* < 0.001, ∗∗∗∗*p* < 0.0001. (For interpretation of the references to colour in this figure legend, the reader is referred to the Web version of this article.)

CNV-Sa have been confirmed in this study to significantly promote cell proliferation, migration, and angiogenesis under normal culture conditions. Given the characteristic hyperglycemic microenvironment of diabetic wounds, we performed a series of functional experiments under high-glucose (HG) conditions herein to clarify the therapeutic potential of CNV-Sa in this condition (Fig. 3A). First, we detected the uptake efficiency of CNV-Sa by HaCaT and HSF under high-glucose conditions (Fig. 3B). Specific red fluorescence was observed in both cell types in this environment, with obvious co-localization with the green fluorescence of viable cells, demonstrating their effective uptake of CNV-Sa even in the hyperglycemic pathological microenvironment. Consistent with the known inhibitory effect of hyperglycemic microenvironment on cell migration, high-glucose treatment (HG group) markedly reduced the migratory capacity of HaCaT, HSF and HUVEC cells (Fig. 3C, Fig. S8). Salidroside monotherapy (Sa group) induced a slight improvement in cell migration that lacked statistical significance. The CNVs group had a certain promoting effect on cell migration. Notably, the non-encapsulated mixture of CNVs and salidroside (CNVs + Sa group) showed only a marginal increase in cell migration, which was not significantly different from that of the CNVs group alone. In contrast, the CNV-Sa treatment group exhibited the most significant migration-promoting effect, increasing the migration rates of HaCaT, HSF, and HUVEC cells to approximately 60.54 ± 1.95%, 54.33 ± 1.21%, and 51.25 ± 5.46% at 24 h, respectively, which were significantly higher than those of other experimental groups (Fig. 3D). These results indicated that CNV-Sa could effectively reverse the cell migration dysfunction induced by high glucose, and the encapsulation of salidroside by CNVs may enhance cellular uptake and stability of the drug, thereby improving its biological activity under high-glucose conditions.

### 3.4 Immunomodulatory potential of CNV-Sa in remodeling the inflammatory microenvironment of diabetic wounds

Chronic diabetic wounds are commonly accompanied by persistent inflammation and immune dysregulation, with prolonged polarization of macrophages toward the pro-inflammatory M1 phenotype considered a major mechanism underlying impaired wound healing [33]. Therefore, regulating macrophage phenotypes and suppressing excessive inflammatory responses are regarded as key strategies to improve diabetic wound repair (Fig. 3E). In this study, the immunomodulatory effects of CNV-Sa were evaluated using an LPS-induced macrophage inflammation model. IL-6, TNF-α, and IL-1β are classic pro-inflammatory cytokines that play crucial roles in the inflammatory response [[34], [35], [36]]. They can recruit more immune cells to the site of inflammation, further amplifying the inflammatory reaction. In contrast, IL-10 is an important anti-inflammatory cytokine that can inhibit the production of pro-inflammatory cytokines and promote the polarization of macrophages toward the M2 phenotype, thereby alleviating the inflammatory response and facilitating tissue repair [37]. At the mRNA level (Fig. 3F), CNV-Sa treatment reduced IL-6, TNF-α, and IL-1β by approximately 13-33% and increased IL-10 about 1.41-fold, indicating a significant anti-inflammatory effect at the transcription level. At the protein level (Fig. 3G), ELISA assays showed that the secretion of pro-inflammatory cytokines exhibited trends consistent with the mRNA data, after CNV-Sa treatment, the protein concentrations of IL-6, TNF-α, and IL-1β decreased by approximately 21-50%, while IL-10 protein levels increased about 2.38-fold, further confirming its anti-inflammatory and immunomodulatory activity. Immunofluorescence analysis further confirmed that LPS treatment enhanced CD86 (an M1 marker) signals and diminished CD206 (an M2 marker) signals, whereas CNV-Sa markedly reduced CD86 expression and increased CD206 expression, indicating a shift of macrophages from a pro-inflammatory to an anti-inflammatory phenotype (Fig. 3H). These findings demonstrated that CNV-Sa could modulate cytokine secretion and macrophage polarization to improve the immune microenvironment of diabetic wounds, providing strong evidence for its potential application in chronic wound healing.

### 3.5 Proteomic analysis of CNV-Sa treated macrophages

**Fig. 4:**
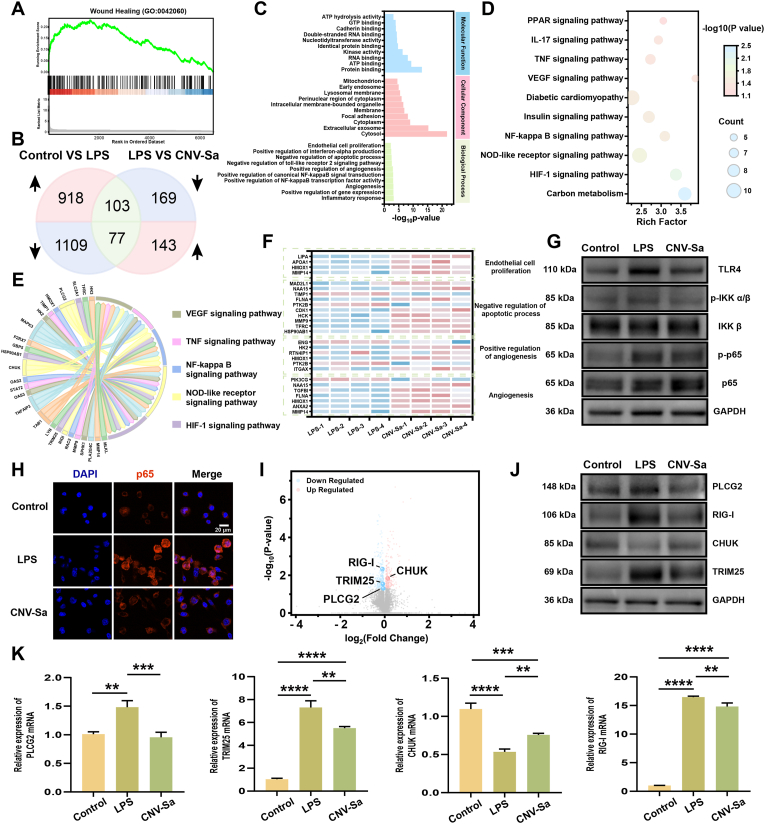
Proteomic analysis of CNV-Sa effects in LPS-induced macrophage inflammation. (A) Gene Set Enrichment Analysis (GSEA) of differentially expressed genes in LPS and CNV-Sa treated groups. (B) Venn diagram showing differentially expressed proteins between LPS and CNV-Sa treatments. (C) Gene Ontology (GO) analysis of differentially expressed proteins involved in immune response, cell proliferation, angiogenesis, and NF-κB signaling. (D) Kyoto Encyclopedia of Genes and Genomes (KEGG) pathway enrichment analysis of differentially expressed proteins. (E) Pathway-gene chord diagram between differentially expressed genes and enriched pathways. (F) Heatmap of differentially expressed proteins related to repair processes. (G) Western blot analysis for TLR4, p-IKKα/β, and p-p65 expression levels in LPS and CNV-Sa-treated groups. (H) Immunofluorescence staining showing p65 nuclear translocation in LPS and CNV-Sa-treated macrophages. Scale bar is 20 μm. (I) Volcano plot of expressed genes between LPS and CNV-Sa group. (J) Western blot analysis of NF-κB pathway-related proteins (PLCG2, RIG-I, CHUK and TRIM25) in LPS and CNV-Sa-treated groups. (K) Quantitative results of gene expression related to PLCG2, TRIM25, CHUK and RIG-I. All n = 3, ∗*p* < 0.05, ∗∗*p* < 0.01, ∗∗∗*p* < 0.001, ∗∗∗∗*p* < 0.0001.

To gain insights into the immunoregulatory mechanism of CNV-Sa in macrophages, we performed a proteomics analysis on THP-1 cells treated with LPS (LPS group) or CNV-Sa + LPS (CNV-Sa group). The acquired data exhibited high completeness (Fig. S9A), with 4857 proteins having 100% completeness (46.31%). After logarithmic transformation, the relative abundance of proteins spanned approximately five orders of magnitude (Fig. S9B–D). Among the 10,488 identified proteins, we first analyzed 204 proteins that were not detected exclusively in the LPS group and 1080 proteins that were identified exclusively in the LPS group (Fig. S9E) and GO analysis indicated that these proteins predominantly participated in diverse critical biological processes (Fig. S9F). The fuzzy clustering analysis on the 4857 proteins commonly identified in all three groups (Fig. S9G) revealed two distinct clusters, whose GO enrichment analysis indicated involvement in basic cellular processes and inflammatory regulation-related pathways (Fig. S9H and I). Gene set enrichment analysis (GSEA) demonstrated that the differentially expressed genes were predominantly enriched in pathways related to "wound healing" (Fig. 4A).

Particularly, we analyzed significantly differentially expressed proteins (DEPs). Among these DEPs, those upregulated by LPS but suppressed by CNV-Sa, as well as those downregulated by LPS but restored by CNV-Sa, were classified as inflammation-alleviating related proteins (Fig. 4B). In contrast, proteins whose expression patterns were consistent with LPS treatment were categorized as inflammation-promoting related proteins (Fig. S9J). Notably, the number of inflammation-alleviating related proteins was significantly higher than that of inflammation-promoting related proteins. GO analysis revealed that these inflammation-alleviating related DEPs were involved in immune response, cell proliferation and angiogenesis signaling, indicating that CNV-Sa not only suppresses the release of pro-inflammatory cytokines but also promotes repair-related signaling pathways (Fig. 4C). KEGG pathway enrichment analysis indicated the potential regulatory effects of CNV-Sa (Fig. 4D–F). The analysis revealed that the differentially expressed proteins were significantly enriched in VEGF, HIF-1, TNF, NF-κB, and NOD-like receptor signaling pathways, which are crucial in immune regulation, angiogenesis, and inflammation. Notably, VEGF and HIF-1 are closely related to angiogenesis [38,39], while TNF and NF-κB are key inflammatory pathways that dominate the inflammatory response in diabetic wounds [40]. Mechanistically, TNF signaling serves as a canonical upstream activator that initiates the NF-κB signaling cascade [41]. Given the central role of the NF-κB signaling pathway in mediating inflammatory cascades and regulating immune microenvironment [42,43], we further focused on the regulatory effects of CNV-Sa on this pathway and its key downstream proteins.

TLR4 served as a primary target receptor for most natural polysaccharides. Upon activation [44], TLR4 induced the activation of multiple downstream transcription factors, including NF-κB and IRF3 [45]. Among the key differentially expressed proteins, we first analyzed the expression profiles of p65, a core component of the NF-κB signaling pathway and its phosphorylated form (p-p65) in THP-1 cells [46]. Upon activated, they directly increase the expression of a variety of inflammatory factors such as TNF-α and IL-6, which are crucial in inflammatory response [47]. Western blot analysis (Fig. 4G, Fig. S10A) showed that LPS treatment markedly upregulated TLR4 expression and elevated the phosphorylation of IKKα/β and p65. These alterations were obviously reversed by CNV-Sa intervention, suggesting that the anti-inflammatory property of CNV-Sa is closely related to NF-κB signaling regulation. Immunofluorescence results further supported this observation (Fig. 4H). On this basis, we further investigated other NF-κB pathway-associated key differentially expressed proteins identified by proteomic screening that are implicated in immune modulation and inflammatory responses (Fig. 4I). RIG-I and TRIM25, which are involved in pattern recognition receptor signaling, were significantly downregulated by CNV-Sa. CHUK (IKKα), a key regulator of the non-canonical NF-κB pathway, was upregulated by CNV-Sa, while PLCG2, another critical NF-κB modulator, was suppressed. These two proteins coordinately modulate the activity of the NF-κB, thus inhibiting persistent pro-inflammatory responses in macrophages (Fig. 4J, Fig. S10B) [48].

At the genetic level, quantitative real-time PCR validation (Fig. 4K, Fig. S10C) verified that the mRNA expression trends were consistent with our proteomic data. By regulating cytokine secretion, macrophage phenotype balance, and inhibiting the NF-κB pathway, CNV-Sa improves the immune microenvironment in diabetic wounds, providing strong evidence for its potential application in chronic wound repair.

### 3.6 Validation of salidroside binding to key proteins by molecular docking, dynamics, and SPR analysis

**Fig. 5:**
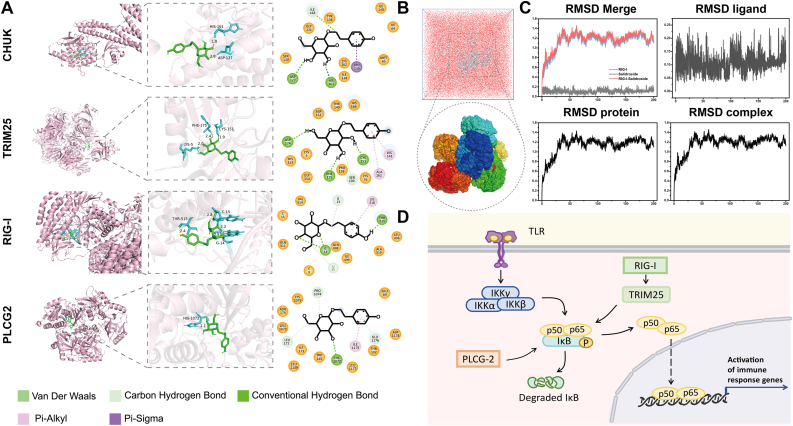
(A) Molecular docking of salidroside with different targets (CHUK, TRIM25, RIG-I and PLCG2). (B) Conformational clustering plot of salidroside/RIG-I complex. (C) Molecular dynamics simulation of salidroside/RIG-I complex. (D) Schematic diagram of the proposed mechanism of the CNV-Sa.

To confirm the direct interaction between salidroside and key regulators of the NF-κB-mediated immune signaling pathway, a combination of computational simulations and experimental validation was performed. Molecular docking simulations predicted that salidroside could bind to the active pockets of four core proteins (RIG-I, PLCG2, TRIM25, and CHUK) involved in this pathway, with detailed molecular interaction patterns illustrated in Fig. 5A. It depicted favorable interaction types including conventional hydrogen bonds, carbon-hydrogen bonds, van der Waals forces, and P-alkyl/π-sigma bonds. Among these target proteins, salidroside exhibited the highest binding affinity for RIG-I, with a docking score of −7.555 kcal mol^−1^, followed by PLCG2 (−6.584 kcal mol^−1^), TRIM25 (−6.325 kcal mol^−1^) and CHUK (−5.965 kcal mol^−1^) (Table S2). As a key thermodynamic parameter reflecting the spontaneity and strength of ligand-protein binding, the docking score (negative binding free energy) indicates spontaneous molecular association under physiological conditions, with more negative values corresponding to stronger and more stable interactions. It was generally recognized that a docking score below −5 kcal mol^−1^ indicated favorable drug-target affinity [49], and the scores of salidroside with all four target proteins fell within this range, confirming the potential of salidroside to interact with these immune regulators. Notably, the remarkably low docking score of salidroside with RIG-I highlighted RIG-I as the primary target of salidroside in regulating immune signaling.

Molecular dynamics (MD) simulations were performed using GROMACS to systematically evaluate the structural stability of four target proteins (RIG-I, CHUK, PLCG2, and TRIM25) in complex with salidroside (RIG-I result shown in Fig. 5B). Over 200 ns simulations, all four systems reached a stable equilibrium state. PLCG2 maintained a smooth Root Mean Square Deviation (RMSD) profile due to its high intrinsic structural rigidity, while CHUK and TRIM25 exhibited moderate conformational fluctuations. Among the four complexes, the salidroside-RIG-I system demonstrated the favorable RMSD trend with the slightest variation (Fig. S11A). Further analysis confirmed that the RMSD values of the salidroside-RIG-I complex and individual RIG-I protein remained stable with negligible fluctuations throughout the simulation, indicating that salidroside binding did not induce significant backbone conformational changes in RIG-I and the complex exhibited good structural stability (Fig. 5C). Radius of gyration (Rg) analysis showed that RIG-I maintained an average Rg value of 5.91 ± 0.07 nm without obvious structural expansion or unfolding, retaining a highly compact conformation (Fig. S11B). Solvent-accessible surface area (SASA) analysis further supported the stability of all four complexes, with consistent surface exposure throughout the simulation (Fig. S11C). Collectively, these computational results suggest that RIG-I is the most promising potential target of salidroside and was therefore selected for subsequent surface plasmon resonance (SPR) experimental validation. The SPR data revealed that salidroside binds directly to RIG-I, exhibiting a distinct fast-binding and fast-dissociation profile with strong dose-dependent affinity. The equilibrium dissociation constant (KD) was 2.77 × 10^−7^ M (Fig. S11D), confirming that salidroside directly targets and binds to RIG-I with high affinity at the sub-micromolar level.

Integrated results from proteomics, Western blotting, qPCR, molecular docking, and molecular dynamics simulation showed LPS stimulation activates TLR signaling triggered IKK complex activation to promote IκB phosphorylation and degradation, leading to nuclear translocation of the NF-κB p50/p65 dimer and transcription of pro-inflammatory genes. CNV-Sa may exert anti-inflammatory effects by targeting RIG-I and other core proteins (PLCG2, TRIM25, CHUK) in this signaling cascade, thereby inhibiting IKK activation, IκB degradation, and NF-κB nuclear translocation to downregulate pro-inflammatory gene expression (Fig. 5D).

### 3.7 Fabrication and characterization of CNV-Sa@GelMA/PVA-MN

**Fig. 6:**
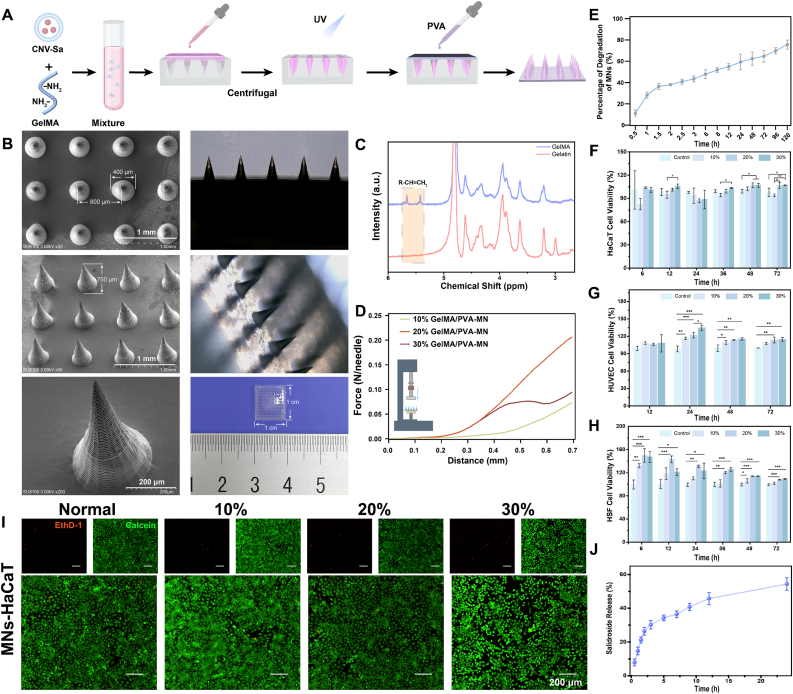
(A) Schematic diagram of the preparation process for GelMA/PVA-MN. (B) SEM and optical microscopy images of GelMA/PVA-MN. (C) ^1^H NMR spectra of GelMA and gelatin. (D) Mechanical strength of microneedles prepared with 10%, 20% and 30% GelMA concentrations. (E) Degradation rate of GelMA/PVA-MN (MNs) in PBS at different time points. (F-H) Cell viability of HaCaT, HUVEC and HSF after exposure to GelMA/PVA-MN for 12-72 h. (I) Live/dead staining of HaCaT after treatment with GelMA/PVA-MN. Scale bar is 200 μm. (J) The sustained release rate of CNV-Sa in CNV-Sa@GelMA/PVA-MN. All n = 3, ∗*p* < 0.05, ∗∗*p* < 0.01, ∗∗∗*p* < 0.001, ∗∗∗∗*p* < 0.0001.

To enhance the penetration and sustained release of CNV-Sa during topical application, we fabricated CNV-Sa into a GelMA/PVA-MN, overcoming the limitations of conventional topical formulations in diabetic wound therapy such as poor tissue penetration and short local retention. The synthesis procedure of GelMA was consistent with our previous study [50]. As shown in Fig. 6A, the preparation involved mixing CNV-Sa with GelMA, centrifugation into molds, UV crosslinking, and finally adding PVA as a backing layer to strengthen the mechanical properties. The microneedles were arranged in a 12×12 array with an 800 μm tip-to-tip spacing between adjacent microneedles. The microneedles exhibited a conical shape with a base edge length of 400 μm and a needle body length of 750 μm, which were suitable for effective skin or wound penetration and enhancing the local delivery of drugs or therapeutic agents (Fig. 6B). The nuclear magnetic resonance (NMR) spectrum in Fig. 6C confirmed the successful synthesis of GelMA, with a clear signal from the methacrylate groups (R-CH=CH_2_) in GelMA, and a substitution degree of 62.59 ± 11.91%.

As clearly shown in the force-displacement curve in Fig. 6D, GelMA/PVA-MN with different concentrations exhibited significant differences in mechanical performance. The force of 20% GelMA/PVA-MN increased continuously and smoothly with penetration depth, reaching nearly 0.20 N/needle at a displacement of 0.7 mm. This value not only exceeded the typical skin insertion threshold of 0.1 N but also showed no signs of fracture throughout the process, demonstrating excellent strength and toughness [51]. In contrast, 30% GelMA/PVA-MN, due to excessive crosslinking, exhibited significantly increased brittleness, with a sudden drop in force indicating fracture occurring at a displacement of approximately 0.4-0.5 mm, which posed a risk of needle-tip residue. Meanwhile, the force of 10% GelMA/PVA-MN remained at a low level and failed to reach the threshold for effective skin penetration even at the maximum insertion depth, indicating insufficient strength. Therefore, 20% GelMA was the safer and more effective concentration for transdermal drug delivery microneedles in this system. A three-point bending test was performed to further assess the deformation behavior and bending properties of the 20% GelMA/PVA-MN substrate. As shown in Fig. S12A, its bending modulus was calculated to be 19.19 ± 3.15 MPa from the force-displacement curve, indicating adequate flexibility to conform well to the skin surface [52]. The flexural strength at break reached 32.16 ± 3.27 MPa, revealing superior structural bending resistance. The 20% GelMA/PVA-MN array could nearly fully penetrate excised mouse skin (Fig. S12B), demonstrating the favorable insertion performance of the microneedles. These results confirm the reversible penetration performance of the microneedles and the excellent biocompatibility of the GelMA/PVA-MN.

Fig. 6E displayed the degradation behavior of GelMA/PVA-MN. In PBS at 37 °C, GelMA/PVA-MN degraded rapidly within the first 1.5 h and then entered a slow degradation phase, with a degradation rate of 75.64 ± 4.23% on day 5. We further assessed GelMA/PVA-MN degradation in a diabetic wound-mimicking medium containing 35 mM glucose (pH 8.0) and 2 μg/mL collagenase I [53]. Higher enzymatic activity accelerated degradation, reaching 94.01 ± 0.86% at 48 h while maintaining a sustained and gradual degradation trend (Fig. S13). Cell viability assays (Fig. 6F–H) confirmed excellent biocompatibility of GelMA/PVA-MN with HaCaT, HUVEC and HSF, maintaining 90-150% of control viability over 12-72 h, with HUVEC and HSF reaching 1.4-1.6-fold higher viability than controls at 24 h. Live/dead staining (Fig. 6I, Fig. S14) further supported these findings, showing strong green fluorescence and minimal red signal across all groups, especially at 20% and 30% GelMA, indicating negligible cytotoxicity.

CNV-Sa@GelMA/PVA-MN was prepared with CNV-Sa incorporated at a protein concentration of 10 μg/mL 3D confocal imaging of PKH26-labeled CNV-Sa revealed uniform distribution throughout the microneedle shaft and tip, with no aggregation or voids, preserving mechanical integrity and penetration efficiency (Fig. S15A and B). In vivo skin insertion results in anesthetized C57BL/6J mice further proved the good minimally invasive property of CNV-Sa@GelMA/PVA-MN (Fig. S15C). Only mild punctate epidermal lesions were observed, which mostly subsided within 12 min and fully vanished within 20 min, without bleeding, edema or acute inflammatory reactions. The in vitro drug-release study in PBS showed that CNV-Sa@GelMA/PVA-MN exhibited a characteristic biphasic release profile (Fig. 6J). A rapid initial burst occurred within the first 3 h, during which the cumulative release reached 30.23 ± 2.45%, largely attributable to diffusion of CNV-Sa located on or near the microneedle surface. This was followed by a sustained and gradual release phase, achieving a cumulative release of 54.32 ± 3.69% at 24 h, indicating that the microneedle network provided effective modulation of the controlled release of CNV-Sa. Overall, CNV-Sa loaded GelMA/PVA-MN possessed good morphology, mechanical strength and biocompatibility, showing great potential for local drug delivery, neovascularization and accelerated diabetic wound healing.

### 3.8 In vivo therapeutic efficacy of CNV-Sa@GelMA/PVA-MN

**Fig. 7:**
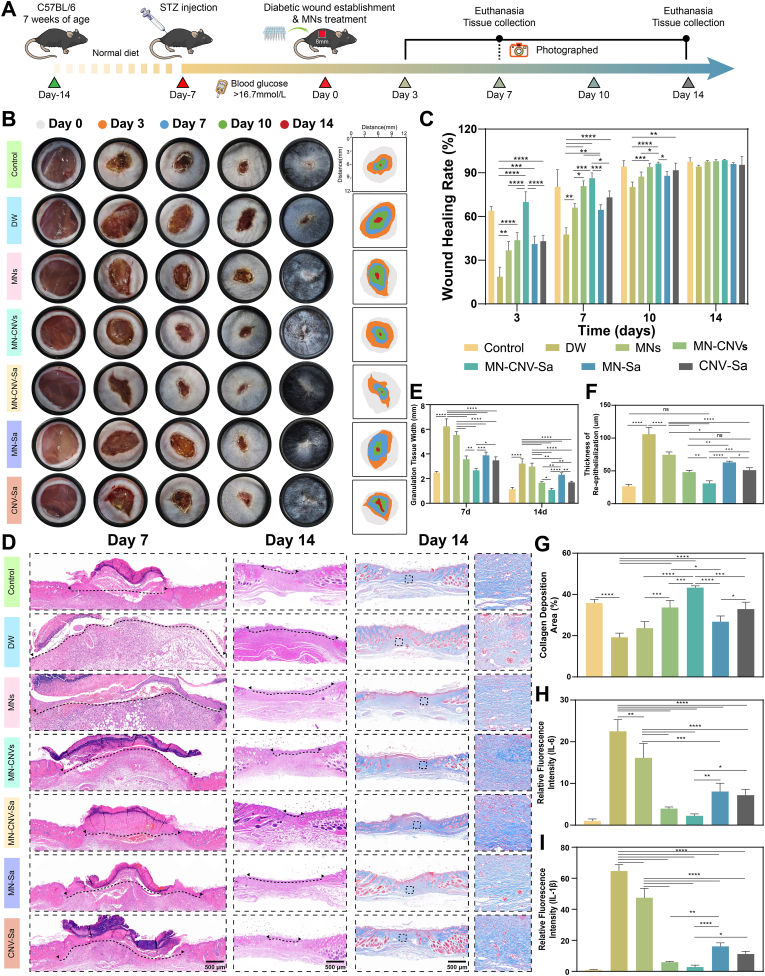
(A) Scheme for diabetic wound healing in mice. (B) Representative photographs of wound healing at different time points post-treatment in each group (Control, DW, MNs, MN-CNVs, MN-CNV-Sa, MN-Sa, and CNV-Sa). (C) Quantitative analysis of wound healing rate at day-0, 3, 7, 10, and 14. (D) H&E and Masson staining of regenerated skin tissue. Scale bar is 500 μm. (E) Quantitative analysis of granulation tissue width among groups. (F) Quantitative analysis of re-epidermal thickness among groups on day 14. (G) Quantitative analysis of collagen deposition area among groups. (H) Relative fluorescence intensity of inflammatory cytokine IL-6 in wound tissues at day 14. (I) Relative fluorescence intensity of inflammatory cytokine IL-1β in wound tissues at day 14. All n = 4, ∗*p* < 0.05, ∗∗*p* < 0.01, ∗∗∗*p* < 0.001, ∗∗∗∗*p* < 0.0001.

Based on the potent in vitro capabilities of CNV-Sa to inhibit inflammation, promote cell proliferation, migration, and angiogenesis, we established a diabetic mouse wound model to evaluate it in vivo therapeutic efficacy (Fig. 7A). Streptozotocin (STZ)-induced diabetic mice have been widely used in studies on diabetic wound healing [54]. To assess the therapeutic effects of different groups, photographs of each group were taken on days 0, 3, 7, 10 and 14 (Fig. 7B). ImageJ software was used to calculate the wound healing rate for effective observation. As shown in Fig. 7C, the wound healing rate reached 64.23 ± 5.05% and 85.72 ± 2.76% of MN-CNV-Sa on days 3 and 7, respectively, which were comparable to those of the control group and significantly higher than those of the MN-CNVs and MN-Sa groups alone. The introduction of the free CNV-Sa group (CNV-Sa) confirmed that the healing efficacy of MN-CNV-Sa was attributed to the transdermal delivery and sustained release properties of the GelMA/PVA microneedle system, rather than the simple application of CNV-Sa alone.

Hematoxylin and eosin (H&E) staining revealed that on day 7, extensive epidermal defects and massive inflammatory cell infiltration were still observed in the DW group and MNs group (Fig. 7D). In contrast, the MN-CNV-Sa treatment group exhibited more complete re-epithelialization process and richer granulation tissue. To provide a comprehensive histological assessment, we further quantified key parameters including residual wound width and re-epithelialization thickness. Quantitative analysis demonstrated that the residual wound width in the MN-CNV-Sa group was significantly narrower than that in the other treatment groups on both days 7 and 14, which reflected its wound contraction-promoting ability (Fig. 7E). The thickness of re-epithelialization on day 14 showed no significant difference between the MN-CNV-Sa and control group, while it was significantly thinner than that in the other treatment groups (Fig. 7F). Masson staining results showed that on day 14, the collagen fibers in the DW group appeared sparse, disorganized, and immature. In contrast, abundant dense and regularly arranged collagen fiber deposits were observed in the dermal layer of wounds in the MN-CNV-Sa group, which morphology was most like the control group. Quantitative analysis of the collagen area percentage confirmed that the collagen deposition rate in the MN-CNV-Sa group was significantly higher than that in the DW group, MNs group, and single-component treatment groups (Fig. 7G). We further assessed angiogenesis on day 14 post-wounding via IHC staining of the vascular marker CD31 (Fig. S16A) and performed quantitative analysis of CD31-optical density (Fig. S16B). The DW and MNs groups presented sparse brown positive signals with poor vascular structure, while the MN-CNV-Sa group showed abundant and well-distributed CD31-positive staining, suggesting superior pro-angiogenic capacity.

To further investigate the anti-inflammatory potential of MN-CNV-Sa in vivo, we used immunofluorescence staining to assess IL-6 and IL-1β expression in wound tissues on day 14. As shown in Fig. 7H–I and S17, the MN-CNV-Sa group exhibited significantly lower relative fluorescence intensities of both cytokines compared to other treatment groups, consistent with in vitro results. These findings indicated that MN-CNV-Sa effectively suppressed inflammation in diabetic wounds, promoting re-epithelialization, collagen deposition, and overall healing. Histopathological examination of major organs (heart, liver, spleen, lung, kidney) confirmed that MN-CNV-Sa caused no obvious systemic toxicity, demonstrating excellent in vivo biocompatibility and safety (Fig. S18).

## 4 Conclusion

This study developed a salidroside-loaded stem cell-derived artificial nanovesicles based microneedle system (CNV-Sa@GelMA/PVA-MN) for diabetic wound treatment and elucidated its therapeutic efficacy and anti-inflammatory mechanism. Functional studies demonstrated that CNV-Sa effectively alleviated cellular inflammation by regulating the NF-κB signaling pathway, while simultaneously promoting the proliferation and migration of skin-related cells and enhancing angiogenesis. The GelMA/PVA microneedle system enabled precise transdermal delivery and sustained local release of CNV-Sa, thereby improving therapeutic efficiency. In vivo experiments further confirmed that CNV-Sa@GelMA/PVA-MN suppressed excessive inflammatory responses and significantly accelerated diabetic wound healing. This microneedle-based therapeutic system represents a promising anti-inflammatory and pro-regenerative strategy for chronic diabetic wound repair.

## CRediT authorship contribution statement

**Junhao Xia:** Conceptualization, Investigation, Methodology, Resources, Writing – original draft. **Fengya Wang:** Conceptualization, Investigation, Methodology, Resources, Writing – original draft. **Yang Song:** Conceptualization, Investigation, Methodology. **Yu Xu:** Conceptualization, Investigation, Methodology. **Mengru Zhu:** Investigation, Methodology, Resources, Writing – original draft. **Wenkun Sun:** Investigation, Methodology. **Yaqi Zhang:** Investigation, Methodology. **Sichun Wang:** Investigation, Methodology. **Qingwen Zhang:** Conceptualization, Investigation, Methodology. **Keman He:** Conceptualization, Investigation, Methodology. **Xin Guan:** Investigation, Methodology. **Hanhan Zhang:** Investigation. **Xiulin Wang:** Methodology. **Lukuan Liu:** Conceptualization, Funding acquisition, Investigation, Methodology, Resources, Supervision. **Jing Liu:** Conceptualization, Investigation, Methodology, Resources, Supervision.

## Declaration of competing interest

The authors declare that they have no known competing financial interests or personal relationships that could have appeared to influence the work reported in this paper.

## Data Availability

The data that has been used is confidential.
