# Supplementary material for: Salidroside-loaded stem cell-derived artificial nanovesicles in hydrogel microneedles alleviate inflammation and enhance diabetic wound regeneration
Source: Mater Today Bio. 2026 Jun 5;39:103323. doi: 10.1016/j.mtbio.2026.103323 (PMC13273594; doi:10.1016/j.mtbio.2026.103323)
Supplement: Multimedia component 1 [file mmc1.docx]

**Salidroside-Loaded Stem Cell-derived Artificial Nanovesicles in Hydrogel Microneedles Alleviate Inflammation and Enhance Diabetic Wound Regeneration**

Junhao Xia^a,1^, Fengya Wang^a,1^, Yang Song^a^, Yu Xu^a^, Mengru Zhu^a^, Wenkun Sun^a^, Yaqi Zhang^a^, Sichun Wang^a^, Qingwen Zhang^a^, Keman He^a^, Xin Guan^a^, Hanhan Zhang^a^, Xiulin Wang^a^, Lukuan Liu^a,*^, Jing Liu^a,*^

^a^ Stem Cell Clinical Research Center, The First Affiliated Hospital of Dalian Medical University, Dalian 116011, China

^1^These authors contributed equally to this work.

*Corresponding authors: Email: lukuanliu@163.com, [liujing@dmu.edu.cn](mailto:liujing@dmu.edu.cn)

**Protein extraction and western blotting.**

Total proteins from CNV-Sa were extracted using RIPA lysis buffer (89901, Thermo Fisher Scientific) supplemented with protease and phosphatase inhibitors (78430 and 78420, Thermo Fisher Scientific). The average protein concentration was measured using the Bicinchoninic acid (BCA) protein assay kit (P0011, Beyotime). Equal amounts of protein samples were subjected to electrophoresis on a 4-12% Bis-Tris gel (MA0463, MeilunBio), using a three-color pre-stained protein standard (AG11919, Accurate Biology) as a molecular weight marker. The separated proteins were then transferred to a 0.2 μm polyvinylidene fluoride (PVDF) membrane (Millipore) for Western blot analysis. The PVDF membrane was incubated with primary antibodies overnight at 4 °C. Subsequently it was incubated with the corresponding HRP-conjugated secondary antibodies at room temperature for 2 hours. The membrane was washed three times with tris buffered saline with tween 20 (TBST) and then developed using an enhanced chemiluminescence (ECL) chemiluminescent reagent. Signal visualization was performed using the Champ Chemi 610 Plus imaging system. The primary antibodies included CD9 (ab236630, Abcam), CD63 (ab134045, Abcam), CD90 (13801S, Cell Signaling Technology), TSG101 (ab125011, Abcam), GAPDH (ab181602, Abcam), TOMM20 (ab186735, Abcam), CANX (2679 S, Cell Signaling Technology), SDCBP (ab133267, Abcam). The secondary antibodies included: Goat anti-Rabbit IgG H&L (HRP) (ab6721, Abcam), Goat anti-Mouse IgG H&L (HRP) (ab6789, Abcam).

**Stability assessment of CNV-Sa.**

The storage stability of CNV-Sa was evaluated by monitoring particle size changes over time under different storage temperatures. Freshly prepared CNV-Sa was aliquoted into multiple tubes and stored at 4 °C, -20 °C, or -80 °C. Samples were collected at predetermined time points, and particle size was measured using NTA. Serum stability was assessed by incubating CNV-Sa in 10% FBS. Prior to use, FBS was ultracentrifuged at 100,000 × g for 120 min at 4 °C to remove endogenous vesicles. CNV-Sa was incubated in the FBS solution at 37 °C for 7 days, and particle size was recorded daily. Freeze-thaw stability was examined by measuring the particle size of CNV-Sa before and after lyophilization followed by rehydration.

**In vitro drug release of CNV-Sa.**

The in vitro release of salidroside (Sa) from CNV-Sa was evaluated by dynamic dialysis. CNV-Sa and free Sa (2 mL each) were sealed in 3.5 kDa dialysis bags and incubated in 10 mL of release medium at 37 °C with 100 rpm shaking. Four release conditions were tested: neutral PBS (pH 7.4, control), acidic PBS (pH 5.5), alkaline PBS (pH 8.0), and high-glucose PBS. At predefined time points, 200 µL of release medium was collected and replaced with fresh release medium. Sa concentration was measured at 274 nm, and cumulative release was calculated based on the initial loaded Sa content.

**Degradation assessment of CNV-Sa.**

CNV-Sa and free Sa solutions containing an equivalent amount of Sa were incubated at 37 °C. Samples were analyzed using UV spectrophotometer to measure absorbance. The Sa concentration and remaining content were calculated based on the standard calibration curve.

**Cytotoxicity Assessment.**

HaCaT, HSF, and HUVEC were seeded separately into 96-well plates at a density of 2×10³ cells per well and incubated at 37 ℃ with 5% CO₂ for 18 hours. Subsequently, vesicles with varying protein concentrations were added to the culture medium. After incubating the cells for different time periods, 10% CCK-8 solution was introduced, followed by an additional 2-hour incubation. The optical density (OD) at 450 nm was then measured.

**Uptake of CNV-Sa.**

To monitor CNV-Sa uptake by HaCaT and HSF cells, we labeled CNV-Sa with PKH26 as per the protocol. After seeding cells, we added the labeled CNV-Sa, stained with Calcein AM and Hoechst 33342, and finally imaged them using a laser confocal quantitative image system.

To investigate the endocytosis mechanism under both normal and high-glucose conditions, HaCaT and HSF cells were seeded into 6-well plates at a density of 2.5 × 10⁵ cells per well. For the high-glucose group, cells were pre-incubated in high-glucose DMEM for 24 h. Thereafter, cells were separately pretreated with different endocytic inhibitors for 1 h at 37 °C: 5-(N-ethyl-N-isopropyl)-amiloride (EIPA, HY-B0285, Med Chem Express, 15μM), methyl-β-cyclodextrin (MβCD, HY-101461, Med Chem Express, 70 μM), and chlorpromazine (CPZ, HY-12708, Med Chem Express, 20 μg/mL). After inhibitor pretreatment, PKH26-labeled CNV-Sa was added and incubated for another 6 h (HSF) or 2 h (HaCaT). Cells were then washed three times with PBS and harvested for flow cytometry analysis.

The cytotoxicity of endocytic inhibitors was assessed by CCK-8 assay. The applied concentrations were set as follows: CPZ (20, 30, 40 μg/mL), EIPA (15, 20, 25 μM), MβCD (70, 80, 90 μM).

**In vitro Scratch Assay.**

HaCaT, HSF, and HUVEC cells were seeded in 24-well plates to form a monolayer. When the cell confluence reached 80-90%, a scratch was made using a sterile 200 μL pipette tip, and floating cell debris was washed away with PBS. Scratch images at 0, 12, and 24 h were captured using an optical microscope, and the wound healing rate was calculated based on the initial and subsequent scratch areas analyzed by Image J.

**Angiogenesis Assay.**

The Matrix-Gel was diluted to 75% with pre-cooled human umbilical vein endothelial cell complete medium and added to the 96-well plate (60 μL/well) on ice, then incubated at 37 °C for 1 h for coating. During this period, P6-P8 HUVEC were digested, counted, and seeded into the coated 96-well plate at a density of 2.5×10⁴ cells/well. After 6 h of co-incubation, HUVEC angiogenesis images were analyzed by Image J.

**Network pharmacology and molecular docking.**

Retrieve the Isomeric SMILES value of salidroside from the PubChem database and download its 3D structure in sdf format. Next, import the Isomeric SMILES into the SwissTargetPrediction and TCMSP bases for target prediction. Additionally, input the compound name into the Comparative Toxicogenomics Database (CTD) to predict potential targets. Unify the predicted targets and import them into UniProt for conversion into standardized gene names. After removing duplicates, compile a list of salidroside’s targets. Perform keyword searches for “wound healing”, “diabetic wound” and “vascularization”, in the GeneCards and OMIM databases to identify diabetic wound related targets. Summarize and remove duplicates to obtain a comprehensive list of diabetic wound-associated targets. Identify the intersection between salidroside’s targets and diabetic wound-related targets. Import these intersecting targets into the STRING database, select “Multiple proteins” and specify “Human sapiens” as the species to generate a protein-protein interaction (PPI) network. Import the resulting TSV file into Cytoscape 3.10.2 for further analysis of the PPI network. Import the core targets into the DAVID database, specifying “Human sapiens” as the species, to perform the GO function and KEGG pathway enrichment analysis. Select significant enrichment results with P<0.01. Analyze the GO and KEGG pathway enrichment diagrams. Finally, the potential salidroside targets related to diabetic wound were imported into Cytoscape 3.10.2 to construct a drug-intersection target-pathway-disease network diagram.

**Molecular dynamics simulation**

The crystal structure of CHUK (PDB: 5TQW), PLCG2 (PDB: 8T7C), RIG-I (PDB: 6KYV), and TRIM25 (PDB: 9IUN) were downloaded from the Protein Data Bank (PDB). Water molecules and redundant ligands in all protein structures were removed via PyMOL. Molecular docking of salidroside against the four target proteins was performed using AutoDock Tools, and the binding conformations were visualized and further analyzed with PyMOL.

The molecular dynamics simulations were conducted using the Gromacs 2022.3 version software in this research. For the pretreatment of small molecules, the AmberTools22 software package was adopted in this study. The small molecules were parameterized with the GAFF force field. The calculated potential data were integrated into the topological file of the molecular dynamics system. The molecular dynamics simulation system was initially subjected to energy minimization via the steepest descent method, followed by a 100,000-step isothermal-isochoric ensemble (NVT) and isothermal-isobaric ensemble (NPT) equilibrium simulations.

**Surface Plasmon Resonance (SPR) Analysis**

Using surface plasmon resonance (SPR) technology , the binding affinity between salidroside and RIG-I was assessed using the Biacore 8K system equipped with a CM5 chip (Cytiva, USA). The activator was prepared by mixing 400 mM 1‐ethyl‐3‐[3‐dimethylaminopropyl] carbodiimide HCl (EDC) (Cytiva, USA) and 100 mM N‐hydroxysuccinimide (NHS) (Cytiva, USA) in a 1:1 ratio and continuously injected at a flow rate of 10 μL/min for 420 seconds to activate the sensor. RIG-I recombinant protein (Ipodix Biological, China) was diluted to 20 μg/mL in sodium acetate buffer (10 mM, pH 4.5) and immobilized on the sample channel (Fc2) at a flow rate of 10 μL/min, achieving an immobilization level of approximately 12,600 response units (RU), the reference channel (Fc1) was left untreated. After immobilization, the chip was deactivated by continuously injecting Ethanolamine hydrochloride (1 M, pH 8 .5) at a flow rate of 10 μL/min for 420 seconds.

Salidroside were diluted to various concentrations (100 - 10000 nm) using 1X PBS-T buffer containing 1% dimethyl sulfoxide (DMSO), each concentration was injected sequentially into channels Fc1-Fc2 at a flow rate of 20 μL/min, with a association phase of 100 seconds and a dissociation phase of 180 seconds. All data were processed and fitted using Biacore Insight software (Cytiva, USA) based on a 1: 1 Langmuir binding model to determine the key binding kinetics parameters association rate constant (ka), dissociation rate constant (kd), and equilibrium dissociation constant (KD) for different concentrations of salidroside and RIG-I.

**Cellular Proteomics Analysis**

The cells used for proteomics analysis were divided into three groups with four biological replicates per group:the Control group consisting of normal cells, the LPS group comprising inflammatory cells after LPS-induced injury, and the CNV-Sa group containing cells treated with CNV-Sa following LPS injury.

Mass spectrometry data acquisition was performed using a SCIEX Triple-TOF 5600+ mass spectrometer, employing a data-independent acquisition mode under positive ionization. The acquisition parameters were set as follows: the scan range for the first-order mass spectrum was 350–1500 m/z with an accumulation time of 0.1 s; for each second-order mass spectrum, the scan range was 100–1500 m/z with an ion accumulation time of 0.04 s. The raw mass spectrometry data were analyzed using DIA-NN 1.9.1 software, searching against the human proteome (downloaded from Uniprot on April 27, 2025) in a library-free mode with default software parameters. Variable modifications were set for methionine oxidation, N-terminal methionine excision, and protein N-terminal acetylation, while amino methylation of cysteine residues was designated as a fixed modification. The "match between runs" function was applied to all analyses, and outputs (precursor ions) were filtered at a false discovery rate (FDR) of 0.01.

**Mechanical and Functional Characterization of MNs.**

The mechanical properties of MNs with varying GelMA tip concentrations were evaluated using a TA+ texture analyzer. MNs were placed tip-up under a 20 mm cylindrical probe, which compressed the needles at 0.05 mm/s to 0.7 mm with a trigger force of 1.5 gf, while force-displacement curves and fracture events were recorded in real time.

For the degradation study of microneedles (MNs), two experimental conditions were set: 1) neutral PBS control group, and 2) diabetic wound-mimicking microenvironment group. For the control group, MNs were immersed in sterile PBS at 37 °C. After reaching maximum swelling (M_0_), samples were continuously incubated. For the diabetic wound-mimicking group, MNs were immersed in a simulated diabetic wound solution containing 35 mM high glucose, pH 8.0 alkaline environment, and 2 µg/mL type I collagenase, with continuous incubation at 37 °C. The solution of the diabetic wound-mimicking system was refreshed every 12 h to maintain the stability of the components in the system. For both groups, at predetermined time points, excess surface water was blotted, and the wet weight (M_t_) was recorded. The degradation rate (%) was calculated as [(M_0_− M_t_)/ M_0_]×100%.

The three-point bending fixture was used to evaluate the bending resistance of the MNs. A piece of 1×1 cm MNs was placed on two supporting spans with a spacing of 7 mm with the needle tips facing upward. A blunt-ended rectangular probe (1 mm in thickness) moved toward the MNs backing at a speed of 1 mm/s with a maximum displacement of 5 mm. The bending modulus was calculated from the initial slope of the force-distance curve, and the flexural strength of the substrate was determined from the peak force value.

To investigate whether the mechanical strength of the MNs was sufficient for skin penetration, an excised mouse skin model was employed. The MNs patch was pressed firmly against the excised mouse skin with the thumb and held steadily for 30 s. The penetrated skin tissue was then stained with trypan blue for 3 min. After removing excess trypan blue, skin images were captured to observe visible blue puncture marks indicating successful stratum corneum penetration.

Microneedle minimally invasive properties were assessed in vivo using C57BL/6J mice. After anesthesia and back hair removal, MNs were inserted and pressed for 30 s, retained for 5 min, then removed. Skin recovery was observed and documented photographically to evaluate tissue restoration after MNs application.


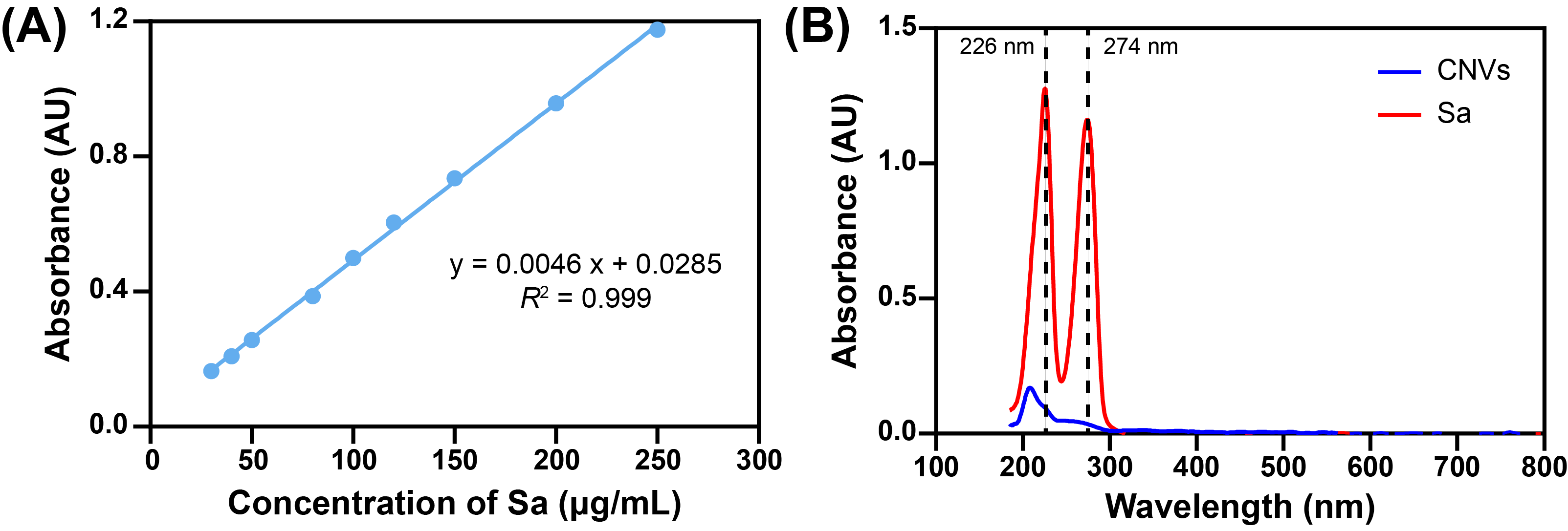


**Figure S1.** (A) Quantitative calibration of Sa concentration by absorbance measurement. (B) UV-Vis absorption spectra of free Sa and blank CNVs.


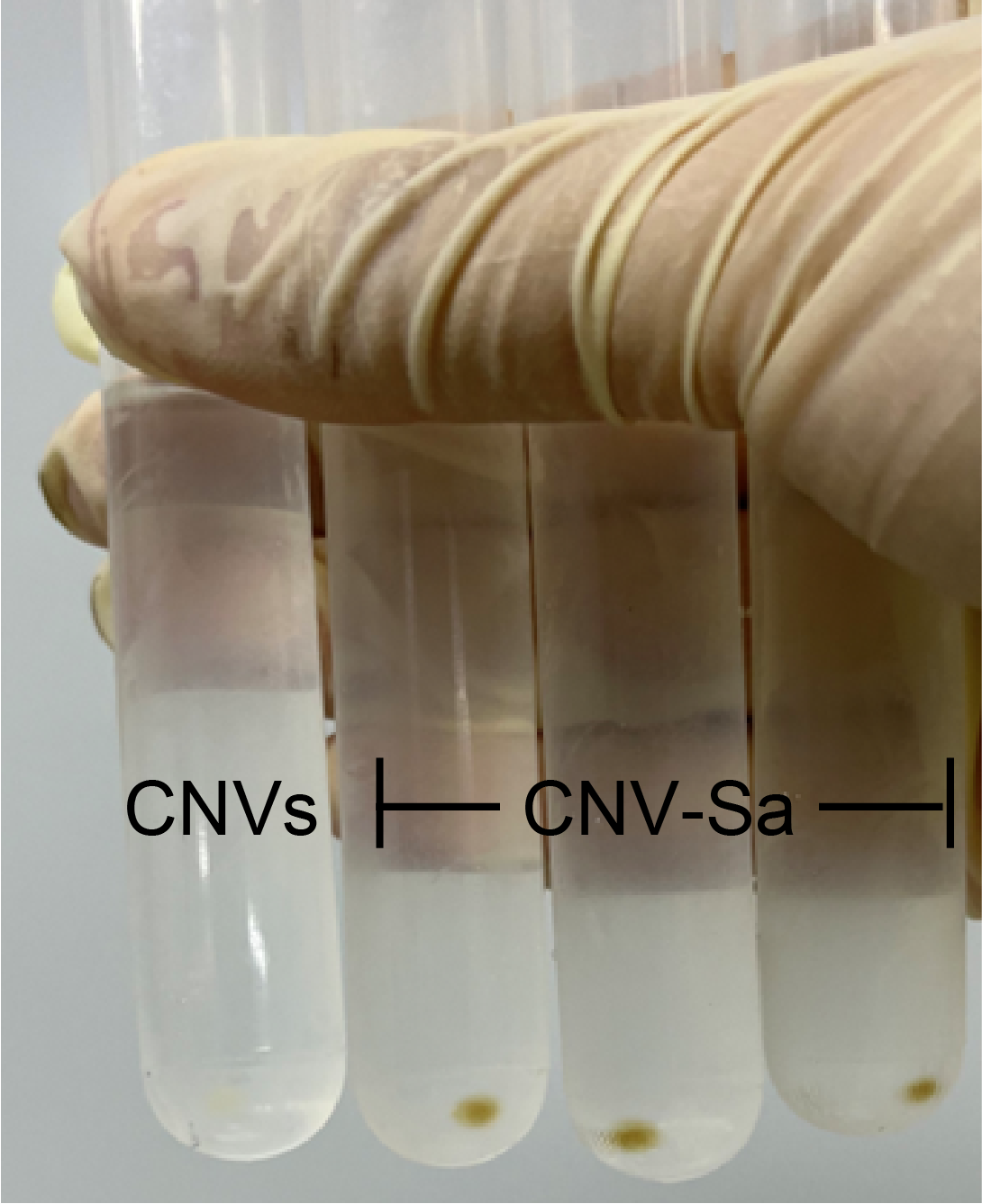


**Figure S2.** Physical image of CNV-Sa.


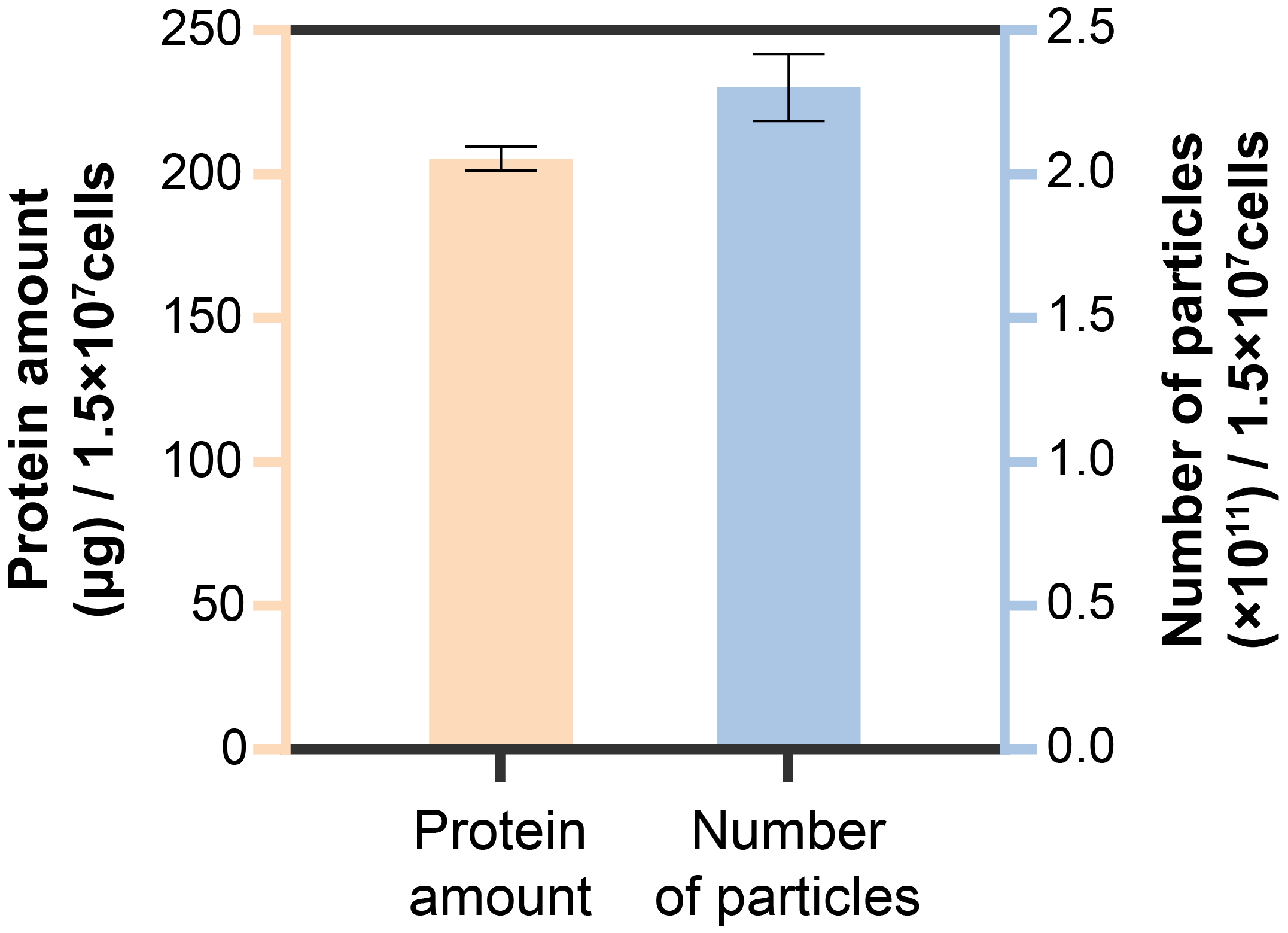


**Figure S3.** Total particle count and protein yield of CNV-Sa per unit cells (n = 4).


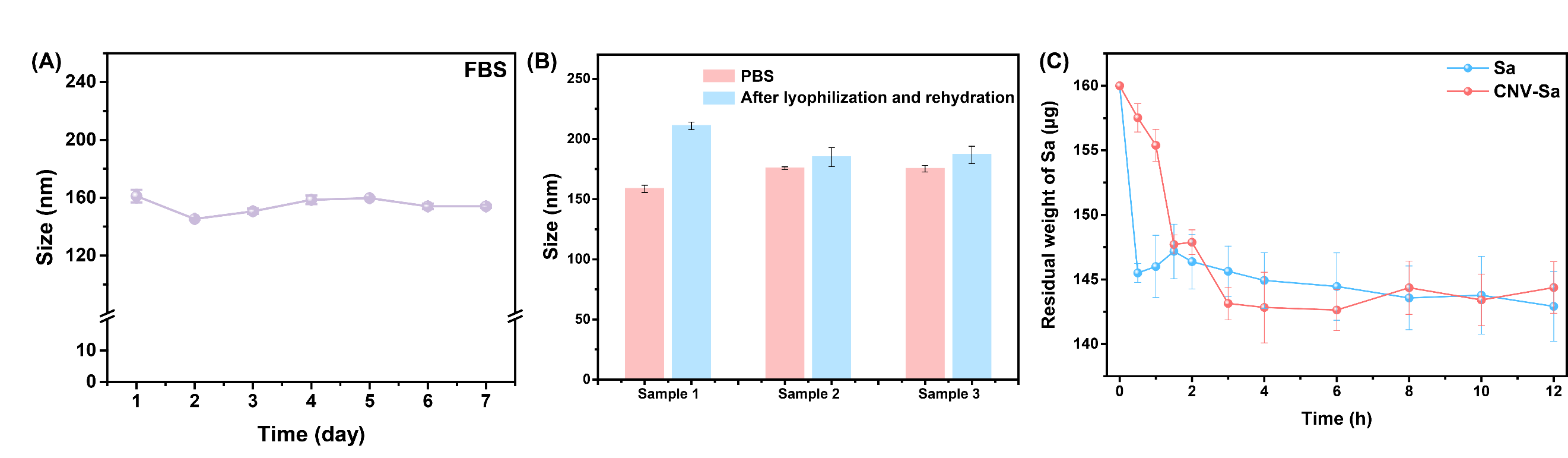


**Figure S4.** (A) Stability of CNV-Sa during incubation in 10% FBS at 37 °C. (B) Particle size comparison of CNV-Sa before and after lyophilization and rehydration. (C) In vitro degradation rate of CNV-Sa. All n = 3.


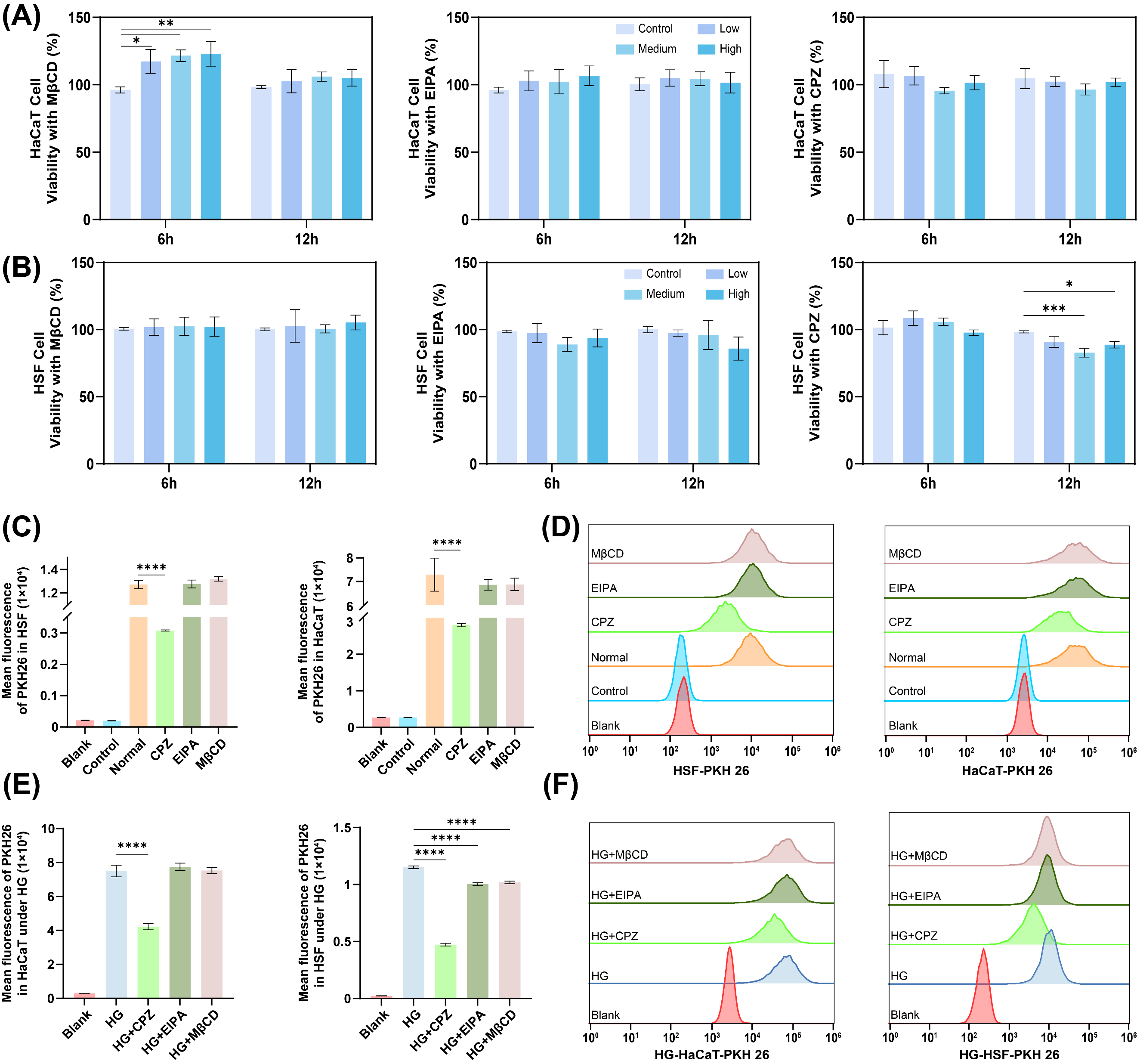


**Figure S5.** (A) Cytotoxicity of inhibitors in HaCaT cells (n = 3). (B) Cytotoxicity of inhibitors in HSF cells (n = 3). (C) CNV-Sa uptake under normal conditions with inhibitors (HaCaT/HSF) (n = 4). (D) Representative flow cytometry histograms (normal conditions). (E) CNV-Sa uptake under high-glucose conditions with inhibitors (n = 4). (F) Representative flow cytometry histograms (high-glucose conditions).


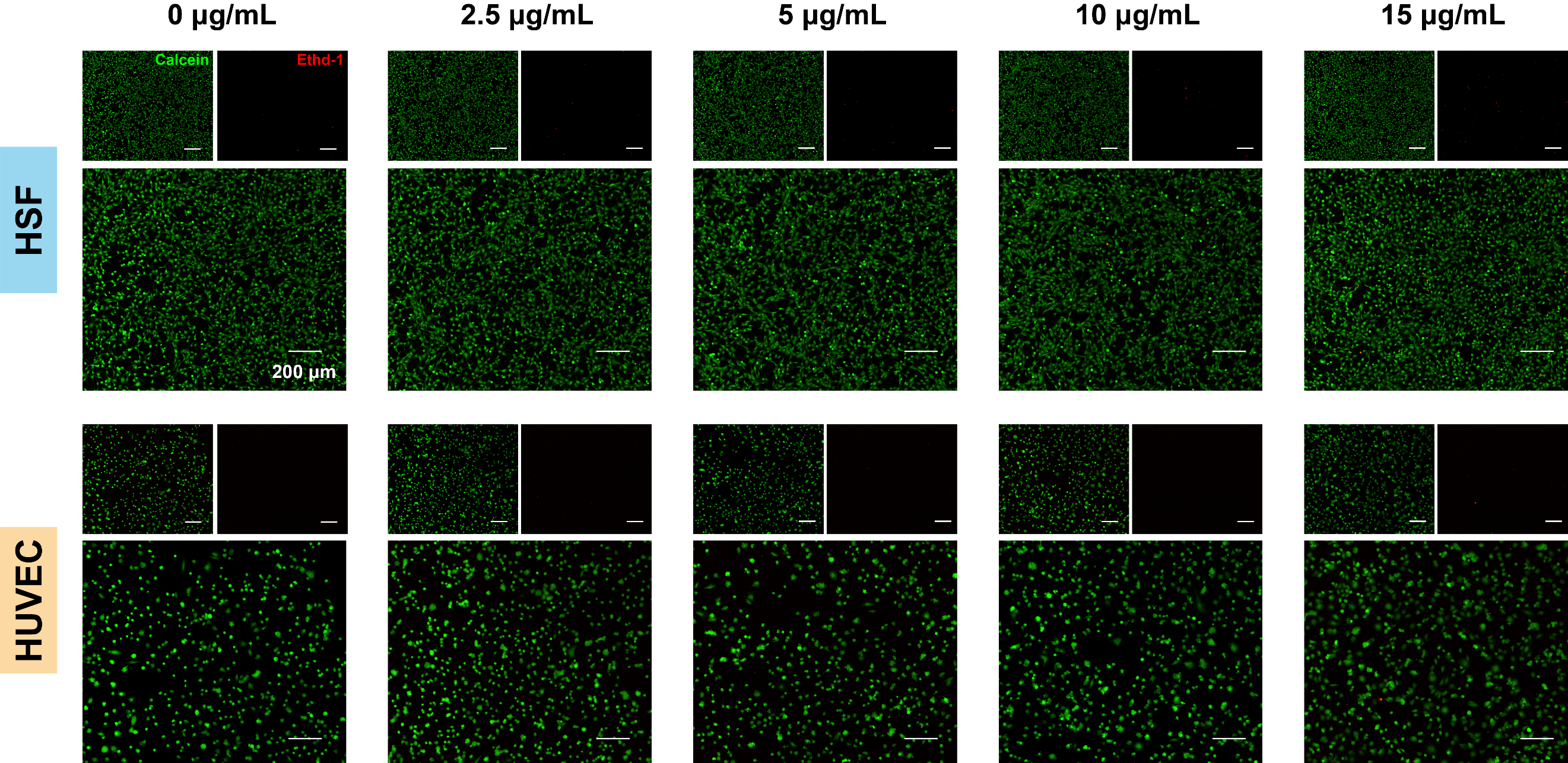


**Figure S6.** Live/dead staining of HSF and HUVEC treatment with different concentrations of CNV-Sa (scale bar = 200 μm).


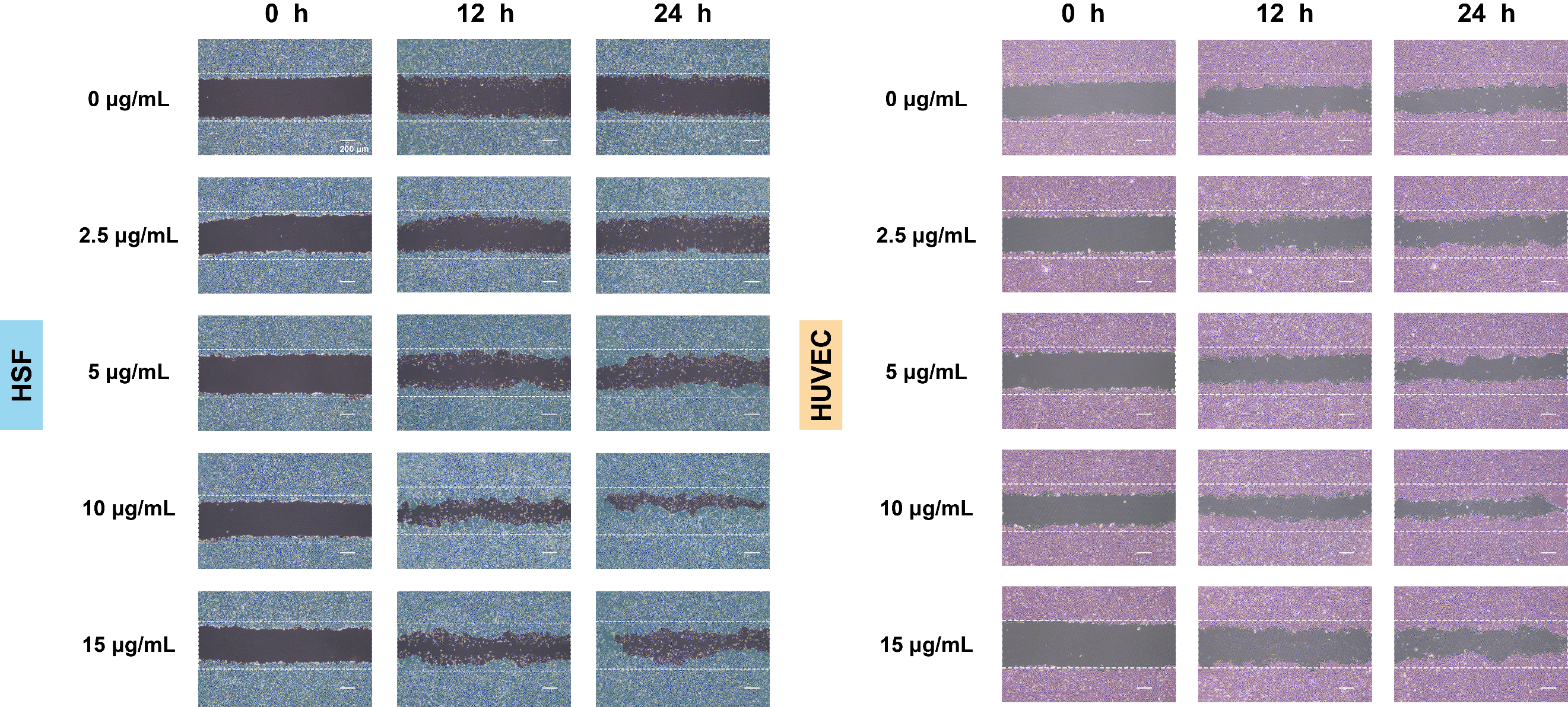


**Figure S7**. Migration capacity of HSF and HUVEC was incubated with CNV-Sa (scale bar = 200 μm).


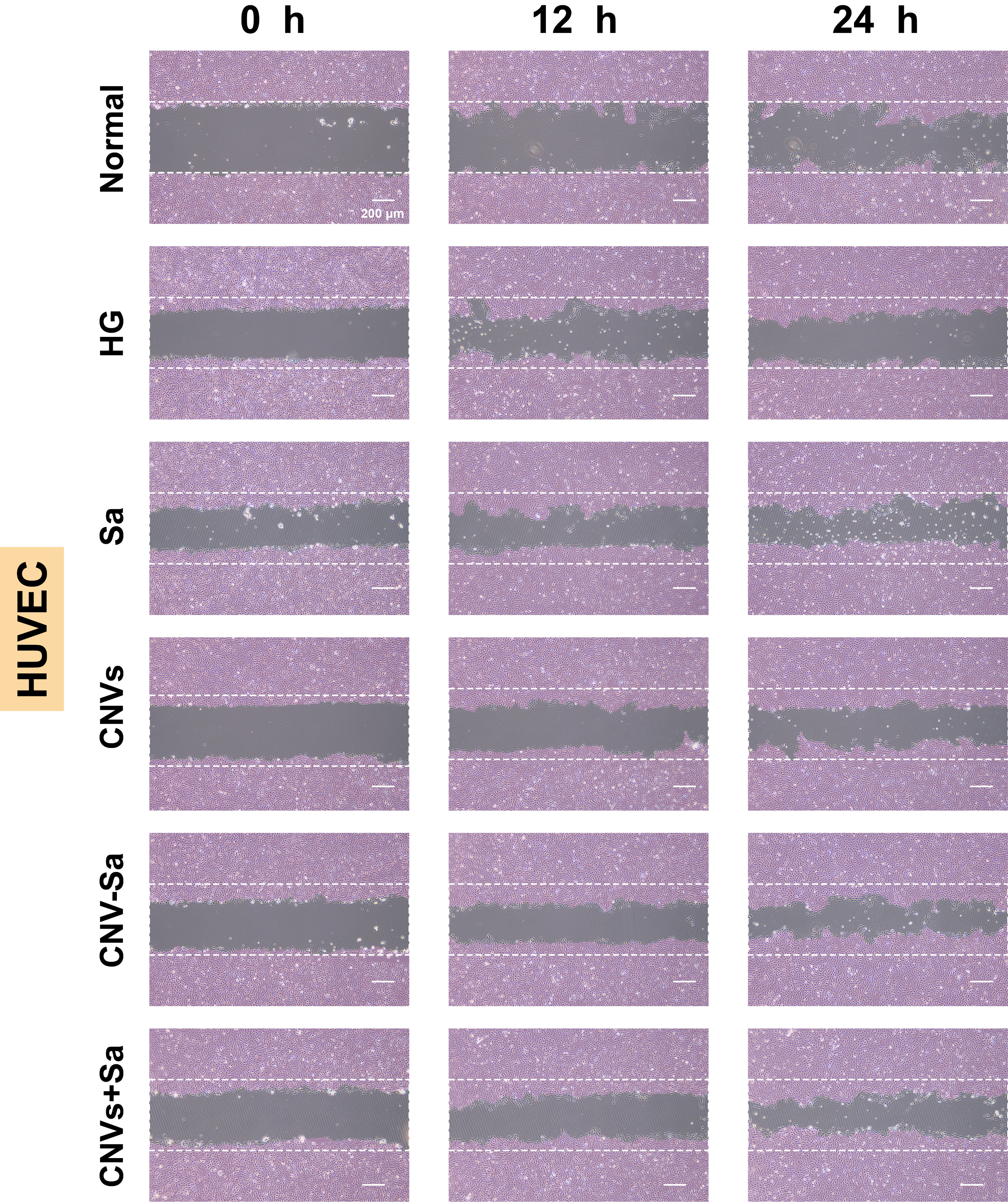


**Figure S8**. Migration capacity of HUVEC was incubated with CNV-Sa under high glucose conditions (scale bar = 200 μm).


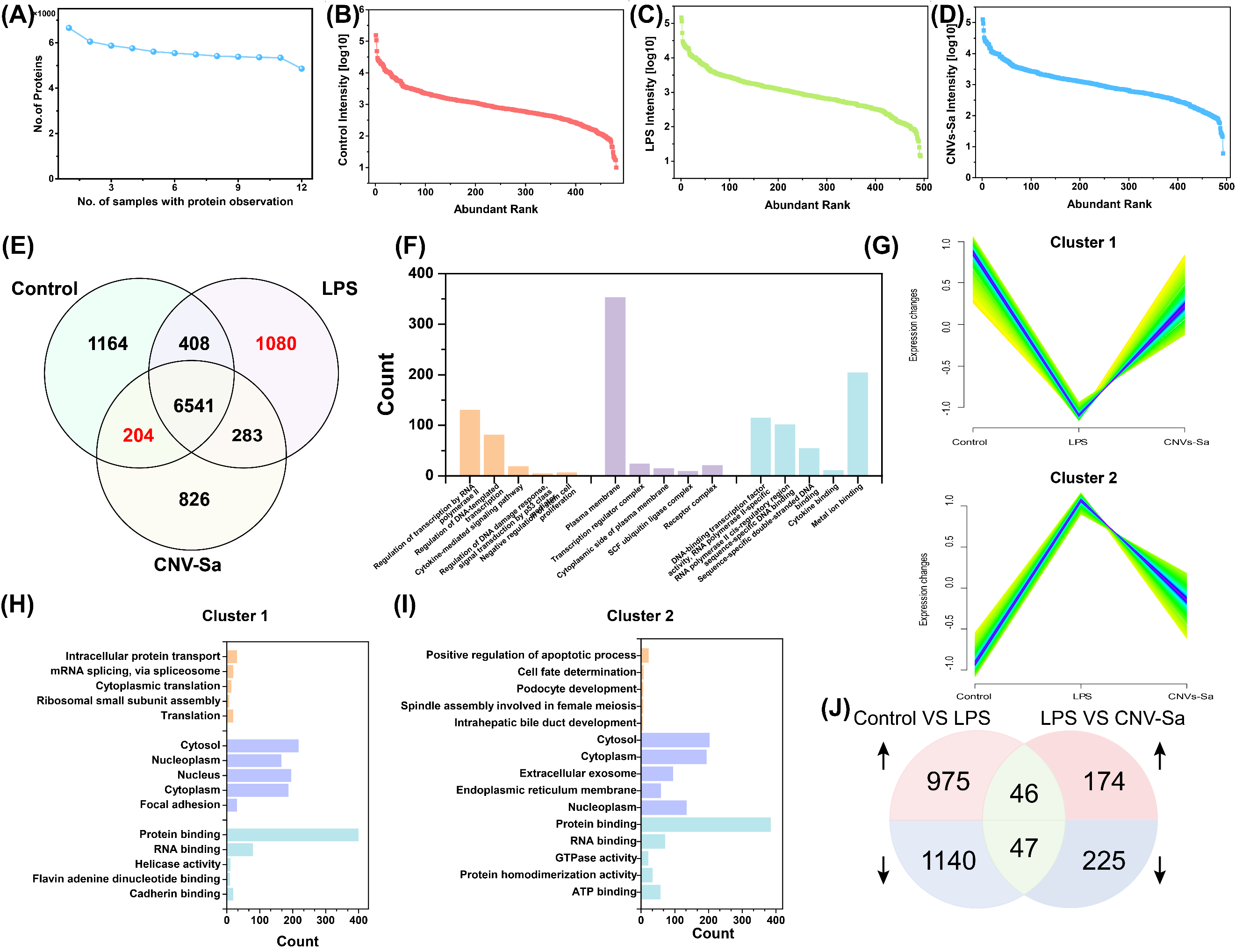


**Figure S9.** Proteomic analysis of CNV-Sa effects in LPS-induced macrophage inflammation. (A) Analysis of the completeness of the omics analysis. (B-D) Sequencing depth analysis of the Control, LPS, and CNV-Sa treated groups. (E) Venn diagram of protein contents of between Control, LPS, CNV-Sa. (F) GO analysis of 1080 elements included exclusively in LPS and 204 common elements in Control and CNV-Sa groups. (G) Mfuzz clustering analysis of the proteins commonly identified in the three groups, selecting protein clusters that were downregulated by LPS but restored by CNV-Sa (cluster 1) and upregulated by LPS but suppressed by CNV-Sa (cluster 2). (H-I) GO analysis of proteins in clusters 1 and 2. (J) Venn diagram consistently expressed proteins between LPS/Control and CNV-Sa/LPS.


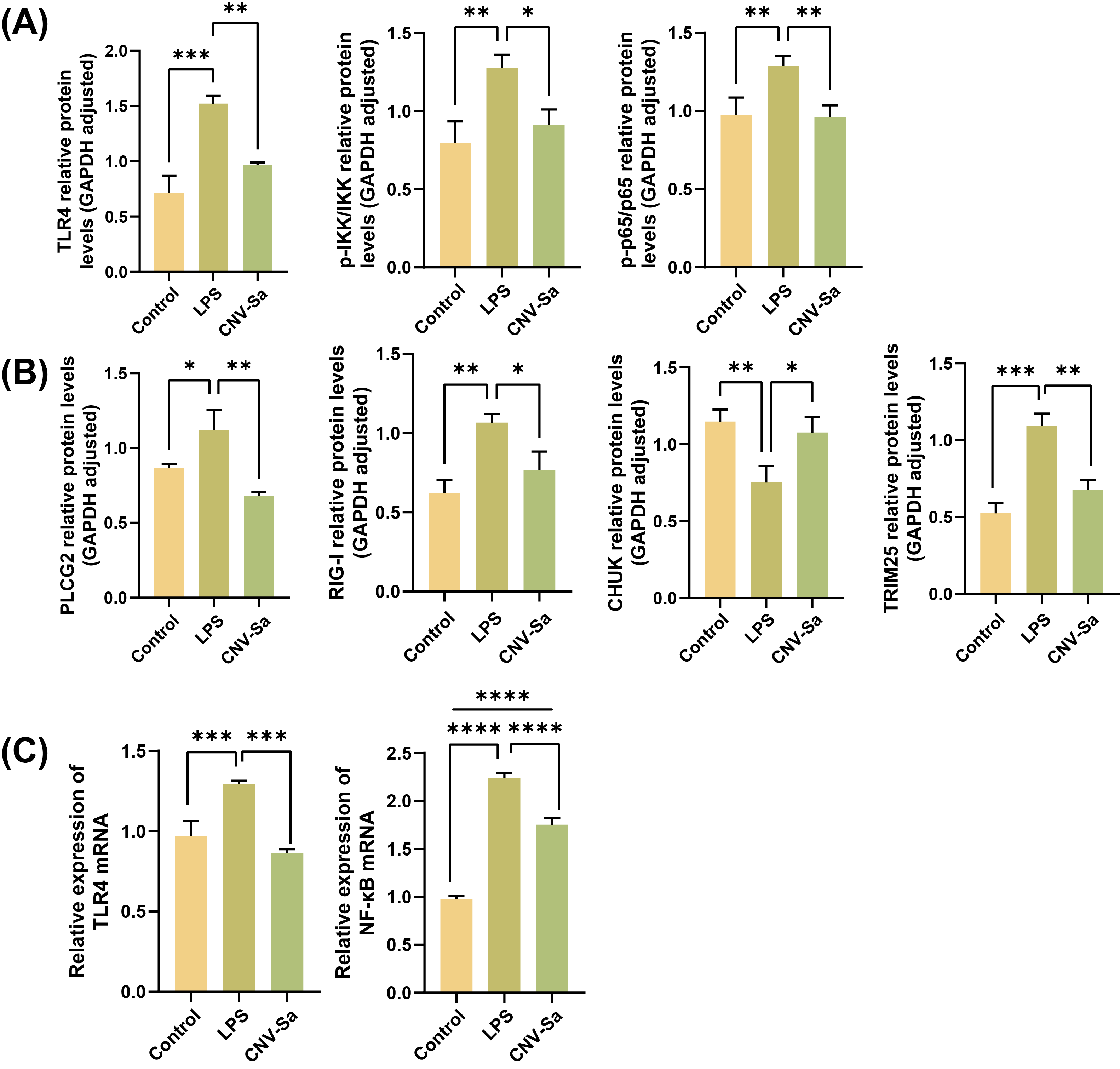


**Figure S10.** (A) The expression levels of TLR4, p-IKK/IκK, and p-p65/p65 in LPS-stimulated macrophages (WB quantification). (B) The expression levels of PLCG2, RIG-I, CHUK, and TRIM25 in LPS-stimulated macrophages (WB quantification). (C) The mRNA expression levels of TLR4 and NF-κB in LPS-stimulated macrophages were suppressed by CNV-Sa. All n = 3.


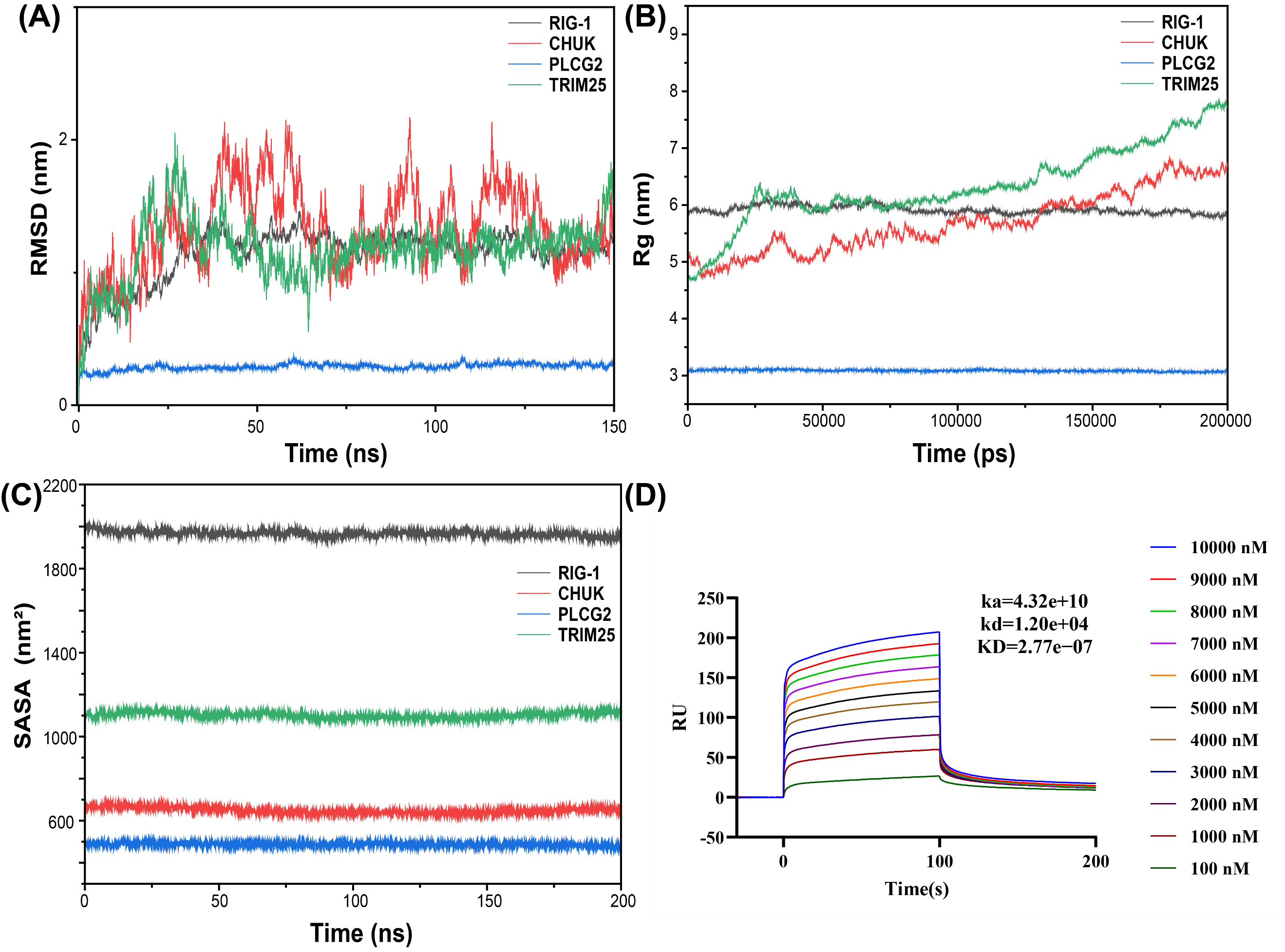


**Figure S11.** (A) Time evolution of the root-mean-square deviation (RMSD) for RIG-I, CHUK, PLCG2, and TRIM25 proteins in complex with salidroside. (B) Time evolution of the radius of gyration (Rg) for the above complexes. (C) Time evolution of the solvent-accessible surface area (SASA) for the above complexes. (D) The binding between RIG-I and varying concentrations of salidroside was assessed by SPR.


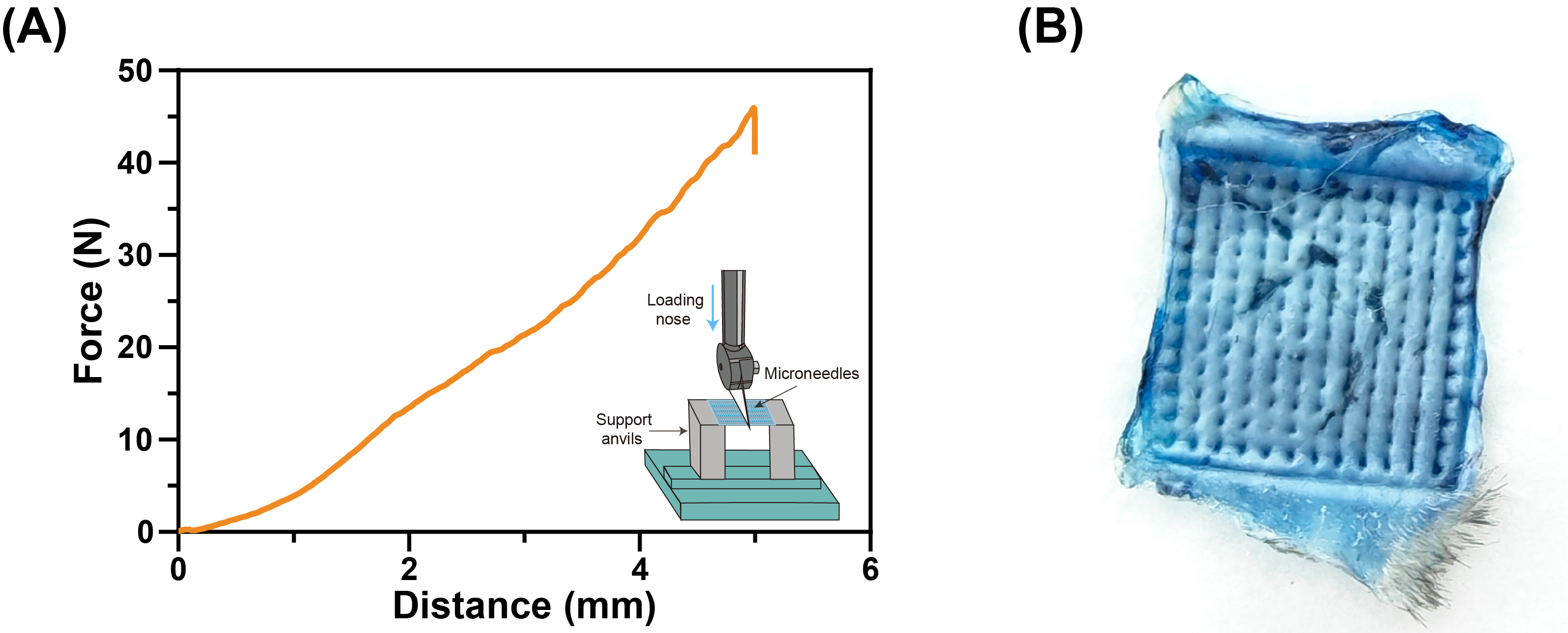


**Figure S12.** (A) The bending properties of the microneedles using a three-point bending setup (n = 4). (B) Ex vivo trypan blue staining of excised mouse skin post-treatment with 20% GelMA/PVA-MN.


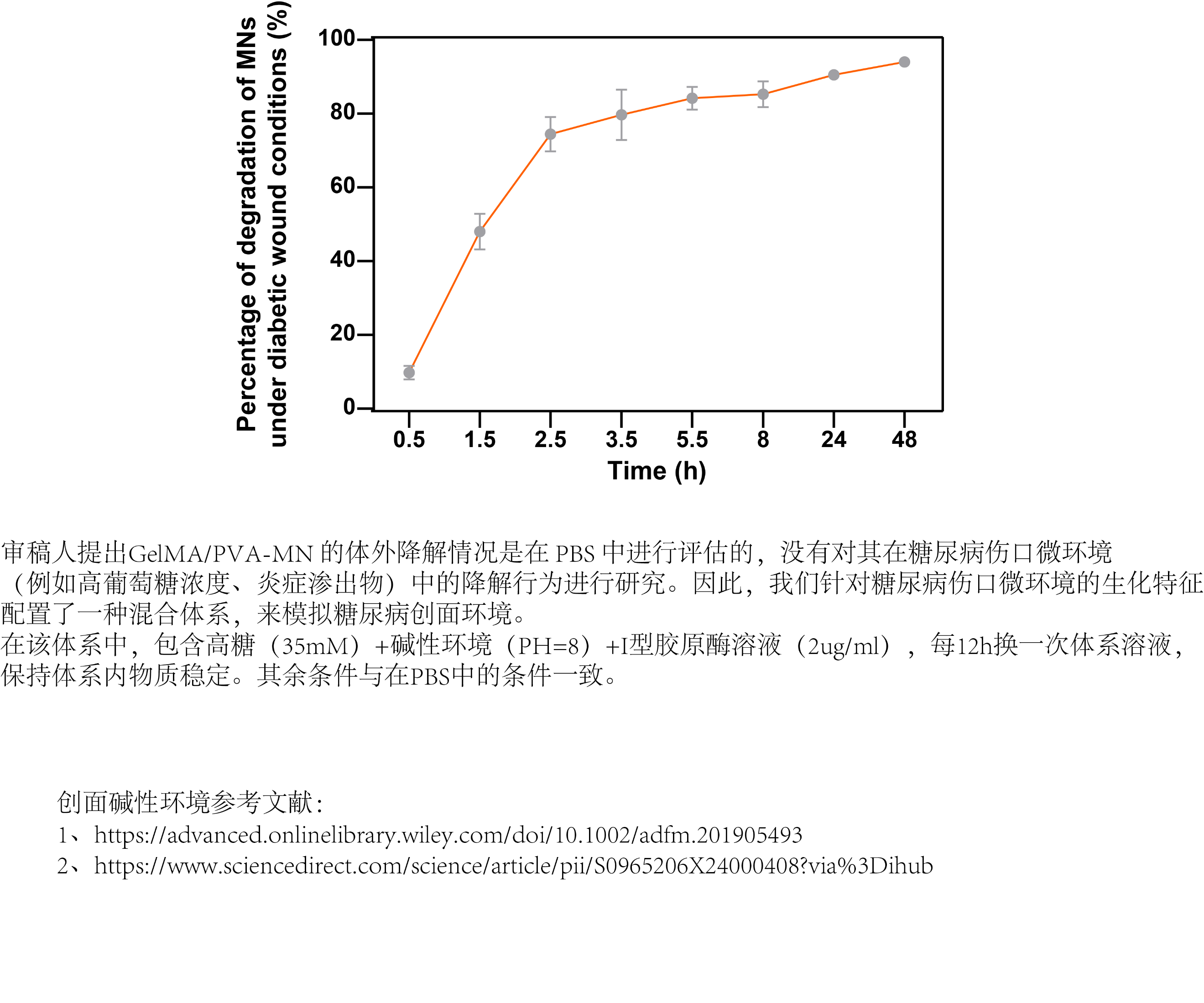


**Figure S13.** Degradation of GelMA/PVA-MN (MNs) under diabetic wound conditions (n = 3).


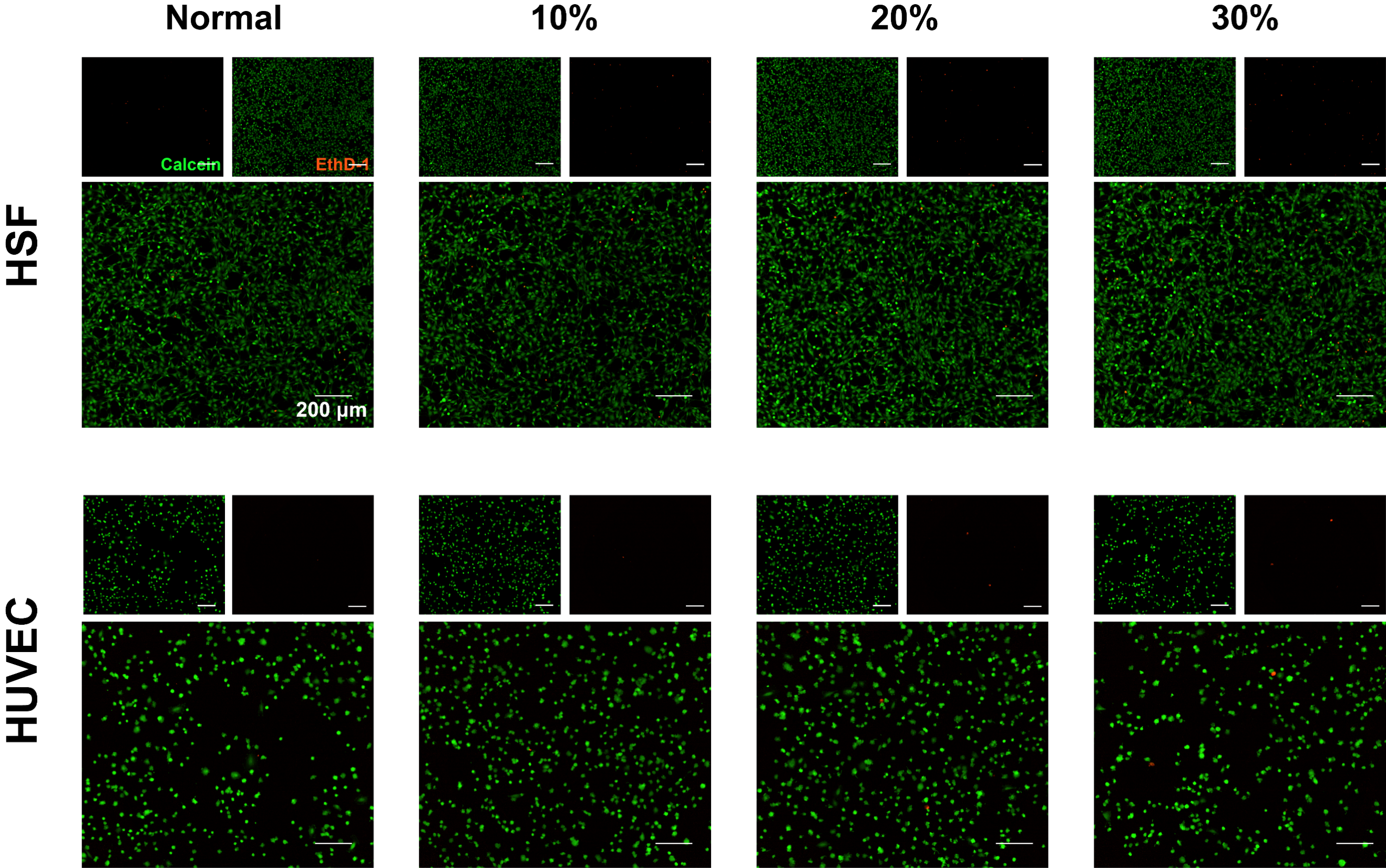


**Figure S14.** Live/dead staining of HUVEC and HSF after treatment with GelMA/PVA-MN (scale bar = 200 μm).


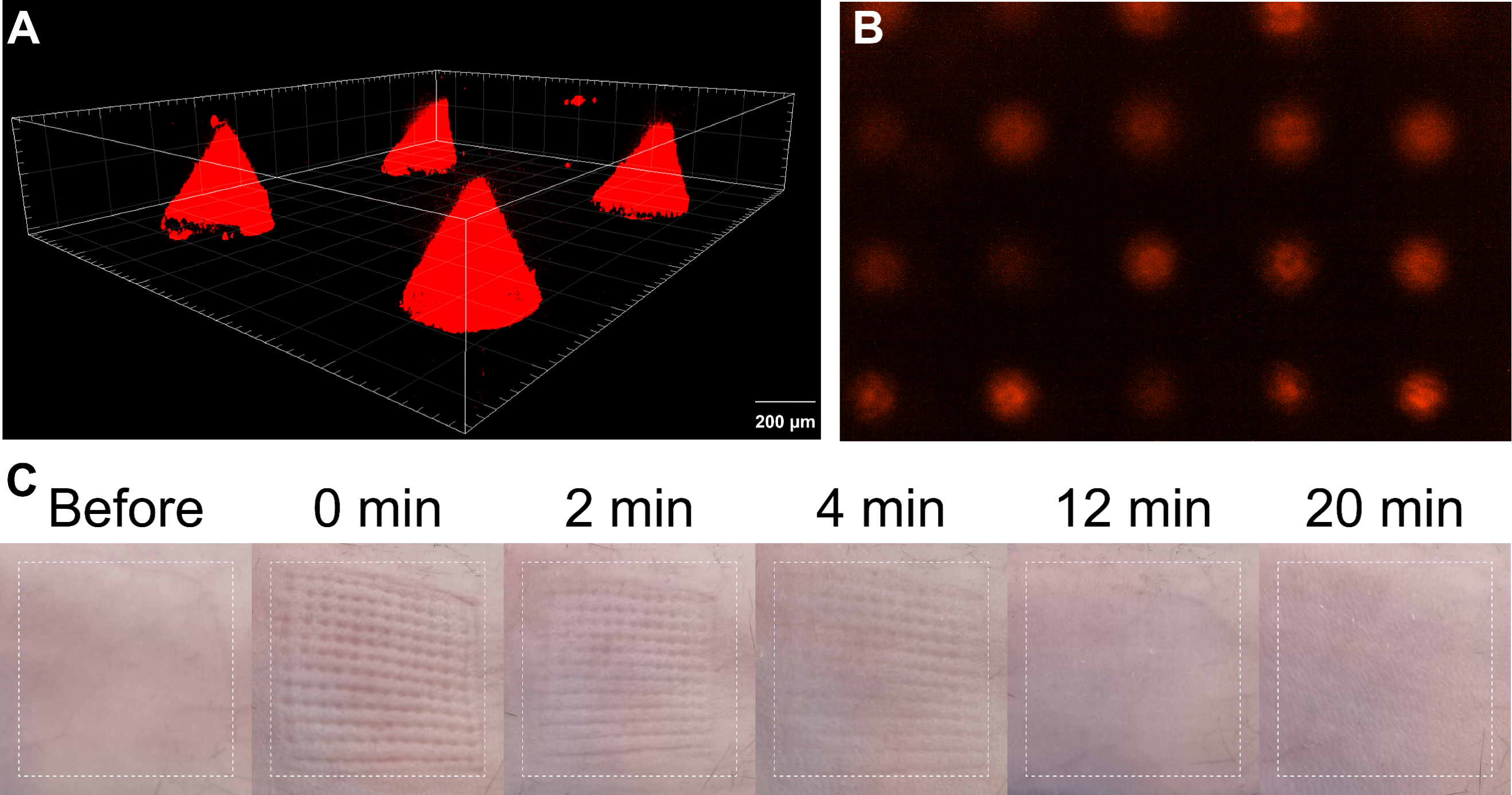


**Figure S15.** (A) 3D Fluorescence distribution image of CNV-Sa in microneedles (scale bar = 200 μm). (B) 3D fluorescence distribution image of CNV-Sa in the XY cross-section of microneedles. (C) In vivo evaluation of minimally invasive skin surface after application of CNV-Sa@GelMA/PVA-MN in mice.


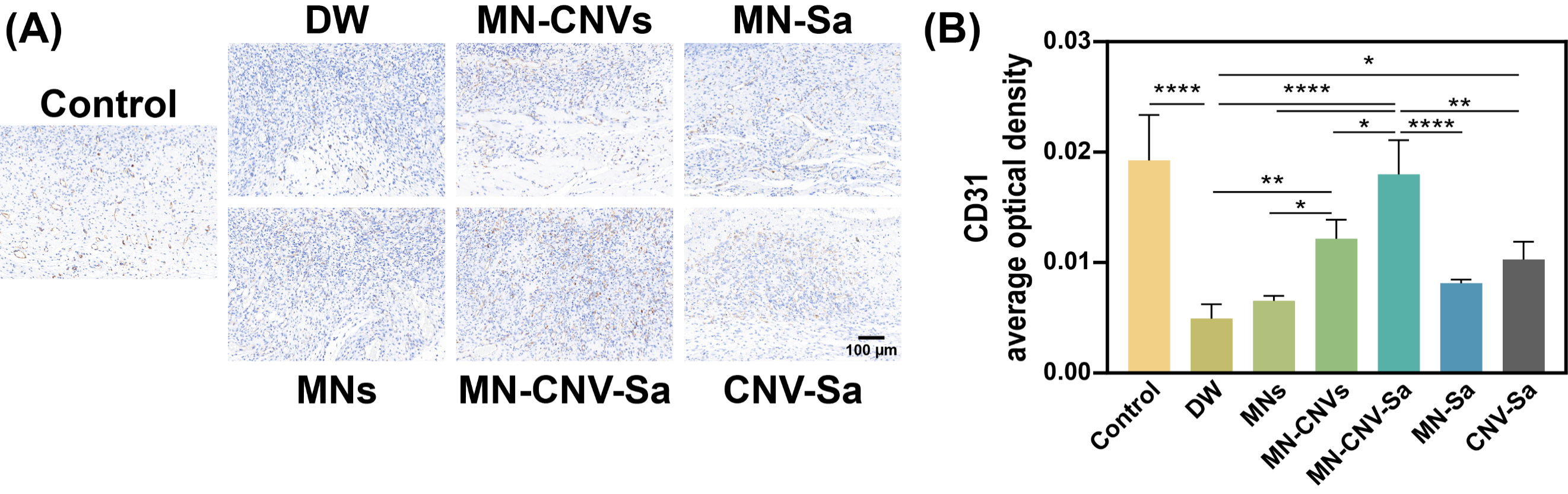


**Figure S16.** Immunohistochemical staining (A) and quantitative analysis (B) of CD31 in wound tissues under different treatments (n = 4, scale bar = 100 μm).


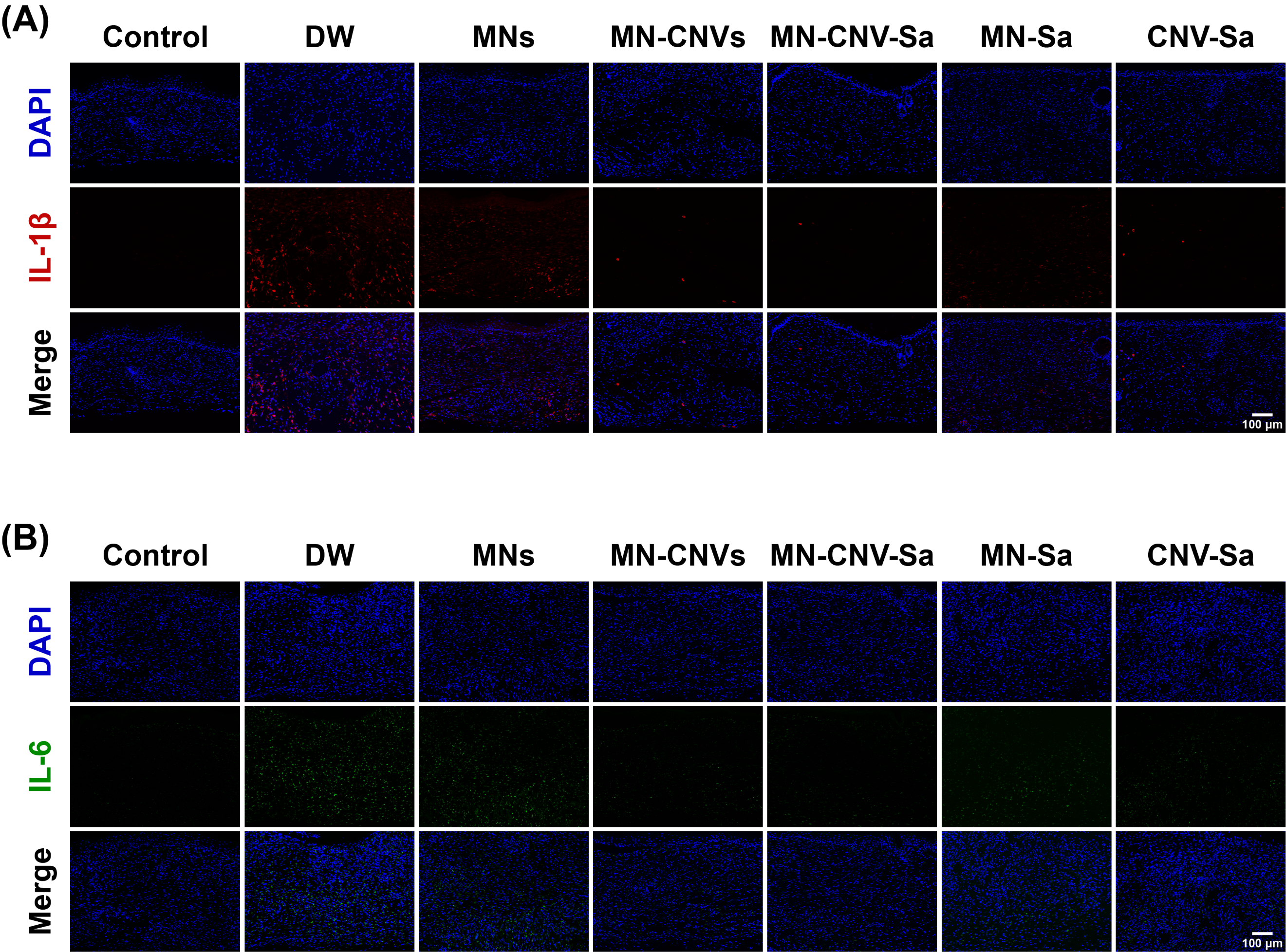


**Figure S17.** Immunofluorescence analysis of IL-1β and IL-6 expression in skin tissue following treatment with Control, DW, MNs, MN-CNVs, MN-CNV-Sa, MN-Sa, and CNV-Sa (n = 4, scale bar = 100 μm).


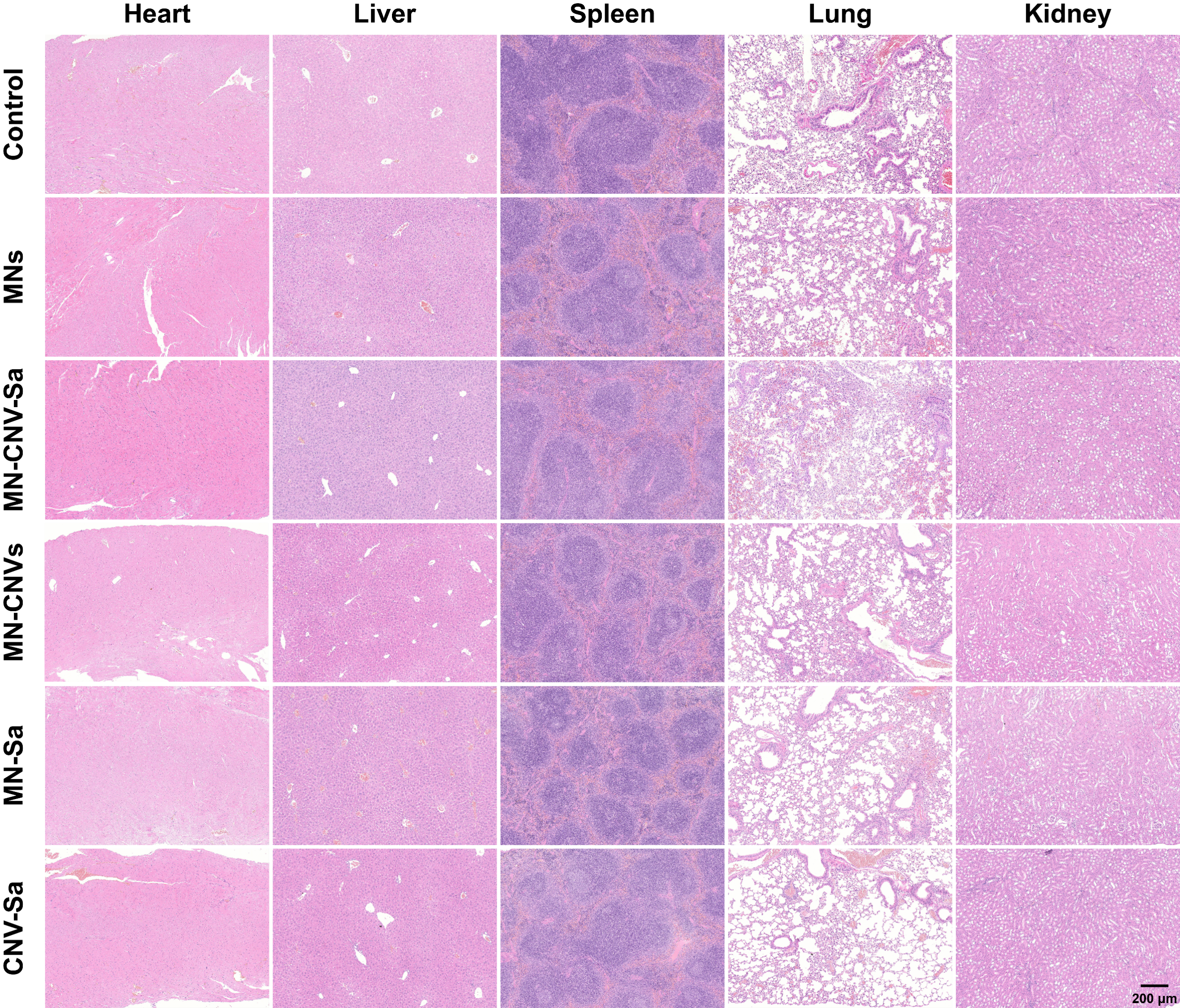


**Figure S18.** Hematoxylin and eosin (H&E) stain images of major organs from diabetic mice following treatment with MNs, MN-CNV-Sa, MN-CNVs, MN-Sa, and CNV-Sa on day 14 (n = 4, scale bar = 200 μm).

**Table S1.** The sequence of PCR primer.

| Gene Name | Primer sequence (forward, reverse) |
| --- | --- |
| β-Actin | F: GGAGATTACTGCCCTGGCTCCTA |
|  | R: GACTCATCGTACTCCTGCTTGCTG |
| IL-6 | F: AGACAGCCACTCACCTCTTCAG |
|  | R: TCTGCCAGTGCCTCTTTGCTG |
| IL-1β | F: CCACAGACCTTCCAGGAGAATG |
|  | R: GTGCAGTTCAGTGATCGTACAGG |
| TNF-α | F: CTCTTCTGCCTGCTGCACTTTG |
|  | R: ATGGGCTACAGGCTTGTCACTC |
| IL-10 | F: GTGATGCCCCAAGCTGAGA |
|  | R: CACGGCCTTGCTCTTGTTTT |
| RIG-I | F: CTGGACCCTACCTACATCCTG |
|  | R: GGCATCCAAAAAGCCACGG |
| TRIM25 | F: AATCGGCTGCGGGAATTTTTC |
|  | R: TCTCACATCATCCAGTGCTCT |
| CHUK | F: GGCTTCGGGAACGTCTGTC |
|  | R: TTTGGTACTTAGCTCTAGGCGA |
| PLCG2 | F: TCCACCACGGTCAATGTAGAT |
|  | R: CCCTGGGCGGATTTCTTTTAT |
| TLR4 | F: AGACCTGTCCCTGAACCCTAT |
|  | R: CGATGGACTTCTAAACCAGCCA |
| NF-κB (p65) | F: ATGTGGAGATCATTGAGCAGC |
|  | R: CCTGGTCCTGTGTAGCCATT |

**Table S2.** Docking scores (kcal mol^-1^) of the salidroside with key proteins.

| Ligand | Target proteins | Scores |
| --- | --- | --- |
| Salidroside | TRIM25 | -6.325 |
|  | CHUK | -5.965 |
|  | RIG-I | -7.555 |
|  | PLCG2 | -6.584 |
